# Sustainable production of myoglobin meat protein in plant chloroplasts

**DOI:** 10.3389/fpls.2026.1876707

**Published:** 2026-08-06

**Authors:** Alexia Groff, Yuhan Lu, Mistianne Feeney, Julian P. Whitelegge, Shengxi Shao, Kyoko Morimoto, Peter Julian Nixon

**Affiliations:** 1 Department of Life Sciences, Imperial College London, London, United Kingdom; 2 Bezos Centre for Sustainable Protein, Imperial College London, London, United Kingdom; 3 Kyomei, Ltd., Cambridge, United Kingdom; 4 Pasarow Mass Spectrometry Laboratory, David Geffen School of Medicine at University of California, Los Angeles (UCLA), Los Angeles, CA, United States; 5 Nanchang University–Imperial College London Joint Laboratory on Photosynthesis and Low Carbon Biotechnology, Key Laboratory of Poyang Lake Environment and Resource Utilization, Nanchang University, Nanchang, China

**Keywords:** *Chlamydomonas*, chloroplast, heme, lettuce, myoglobin, tobacco, transplastomic

## Abstract

Alternative routes for producing animal proteins are crucial for reducing the reliance on traditional livestock farming, which contributes significantly to greenhouse gas emissions, deforestation, and water consumption. Myoglobin (Mb) is an important oxygen-binding hemoprotein found in vertebrate muscle which enhances the nutritional and sensorial properties of meat. Due to its unique functionality, Mb has been heterologously expressed in a variety of organisms, although only transient expression in *Nicotiana benthamiana* has been reported for higher plants. In this study, we used chloroplast transformation technology to express porcine Mb in higher plants (tobacco, a non-edible model plant, and lettuce, an edible host) and bovine Mb in the green alga *Chlamydomonas reinhardtii*. Mb accumulation was estimated by immunoblotting and found to be much higher in tobacco (2.7% total soluble protein (TSP)) and lettuce (1.5% TSP) than *Chlamydomonas reinhardtii* (<0.25% TSP). The expression in tobacco chloroplasts is also superior to tobacco nuclear expression (using either the cauliflower mosaic virus 35S promoter or the *Arabidopsis thaliana* ubiquitin promoter). Total heme levels were elevated in myoglobin-producing mutants compared with control plants, although porcine Mb purified from tobacco leaves exhibited approximately 35% heme-binding (compared with 80% heme-binding in *E. coli*-expressed Mb), despite being correctly folded, suggesting that heme availability might be a bottleneck. Overall, our work describes the first report of stable Mb production in higher plants and its effect on photosynthesis and heme levels. This provides a foundation for future plant-made animal proteins for food applications.

## 1 Introduction

Traditional animal agriculture is a resource-intensive and ethically contentious practice, which contributes significantly to greenhouse gas emissions, deforestation, and biodiversity loss (Koneswaran and Nierenberg, 2008; Anomaly, 2015). In contrast, alternative protein sources like plant-based proteins can provide a more sustainable and scalable solution to meet the increasing demand for protein-rich foods (National Academies of Sciences, Engineering, and Medicine, 2023), especially in a world where food security is increasingly strained by global population growth, climate change, and crop diseases (Lurie-Luke, 2024).

Nutritionally important meat proteins can also be produced by alternative methods, whilst maintaining similar nutritional profiles and functional properties to traditional meat (Carlsson et al., 2020). One such protein is myoglobin (Mb), a 17-kDa oxygen-storage hemoprotein found in cardiac and skeletal muscle of vertebrates, which is composed of a single polypeptide chain, folded into eight alpha helices, that binds a single *b*-type heme (Kendrew et al., 1958). The central iron cation of the heme can make six coordinate bonds: four to the nitrogen atoms of the heme’s porphyrin ring, one to the proximal histidine group of the myoglobin chain (H93), and one to a ligand such as oxygen (Suman and Joseph, 2013). The ligand bound at this position determines the perceived colour of muscle, and therefore meat; for example, oxygen (“oxymyoglobin”) gives meat a bright-red colour, whereas absence of ligand (“deoxymyoglobin”) gives meat a purple-red colour (Suman and Joseph, 2013).

Heme is also an important source of bioavailable iron since it is more easily taken up by the body than non-heme iron, which is much more abundant in plants than heme iron (Fairweather-Tait, 2023). Mb also plays a key role in imparting a “metallic” and umami flavour to meat (Suman and Joseph, 2013). For these reasons, Mb is a particularly interesting product in conferring meat-like properties to meat alternatives; for example, the plant-based food company “Impossible Foods” has recombinantly expressed a soy root nodule phytoglobin known as leghemoglobin in the yeast *Komagataella pastoris* for incorporation into their alternative meat products (Santo et al., 2020). Other heterotrophs used for Mb expression include *Escherichia coli* (Varadarajan et al., 1985; Cutruzzolà et al., 1996; Meng et al., 2023) and *Saccharomyces cerevisiae* (Shimada et al., 1989; Xue et al., 2023); however, heterotrophic protein production is currently limited in terms of scalability and reliance on expensive and energy-intensive bioreactors and feedstocks (Jareonsin and Pumas, 2021).

In the case of plants, Mb expression has been reported in the leaves of *Nicotiana benthamiana* (a relative of cultivated tobacco) by transient expression using a viral vector delivered by *Agrobacterium tumefaciens* (Carlsson et al., 2020). Although constructs were designed for both cytosol-targeted and chloroplast-targeted human Mb, chloroplast-targeted Mb was abandoned at an early stage due to protein size heterogeneity observed by SDS-PAGE. The cytosol-targeted Mb was purified and demonstrated both heme incorporation and protein functionality. However, transient expression is constrained in scalability for sustainable food protein production. As for stable expression, chloroplast transformation of plants offers several advantages over nuclear transformation, mainly high transgene expression thanks to the prokaryotic ancestry of the chloroplast, high copy number, and lack of gene silencing mechanisms (Adem et al., 2017). Other advantages include limited environmental dispersion of the transgene thanks to the maternal inheritance of the chloroplast and the lack of a positional effect on gene expression since integration occurs by homologous recombination into a specific site in the chloroplast genome (Adem et al., 2017).

The chloroplast is an interesting site for Mb expression as it is also the site of heme (iron-containing compound of the porphyrin class) production, Mb’s prosthetic group (Chen et al., 2024). This pathway, known as the tetrapyrrole pathway, is one shared between the four classes of tetrapyrroles found in plants: heme, chlorophyll, siroheme, and phytochromobilin (Tanaka and Tanaka, 2007). There are also important feedback mechanisms in this pathway which regulate tetrapyrrole production (Tanaka and Tanaka, 2007).

Regarding the transplastomic expression of Mb, there has been one report of chloroplast transformation: the unicellular alga *Chlamydomonas reinhardtii*, specifically the cell wall-less strain CC-5168 (*psbH* mutant TN72), was transformed to express N-terminally FLAG-tagged bovine Mb with the aim of culturing the Mb-enriched biomass as a meat alternative (Phadnis and Prakash, 2024). Mb accumulated to ~0.234 mg/g of dry cell weight in 2-L cultures, still significantly suboptimal compared with animal meat (e.g., 6–7 mg/g for beef) (Phadnis and Prakash, 2024). Despite plant chloroplasts generally having higher transgene expression than algal chloroplasts (Rasala et al., 2011), there are currently no reports in the literature of stable transformation of higher plants for Mb accumulation.

Here, we report the production of Mb via chloroplast transformation of the model plant *Nicotiana tabacum* (tobacco), appreciated for its high transgene expression and ease of transformation (Daniell et al., 2002; Castiglia et al., 2016), and of *Lactuca sativa* (lettuce), a cultivated crop that is also amenable to chloroplast transformation (Kanamoto et al., 2006; van Eerde et al., 2019). As far as the authors are aware, this is the first report of heterologous hemoprotein expression in lettuce. For comparison, we also expressed Mb in these plants by nuclear transformation without specific subcellular targeting and in the chloroplast of *Chlamydomonas reinhardtii*. Porcine and bovine Mb were expressed due to their relevance to the food industry; porcine Mb specifically has a lower susceptibility to autooxidation compared with other orthologs (Suman and Joseph, 2013). Levels of Mb accumulation were quantified, and purified Mb was assessed for heme binding, oxidative damage, and protein folding. The impacts of expressing a heme-binding protein on the photosynthetic performance and on the levels of heme *in planta* were also assessed.

## 2 Materials and methods

### 2.1 Plant and algal growth conditions

Sterilised seeds of *N. tabacum* cv. Petit Havana (provided by Dr. Marta Hojka) and *L. sativa* line sK23 (provided by Kyomei Ltd.) were sown on media containing Murashige and Skoog (MS) salts (Skoog and Miller, 1957) (Sigma) with 3% (w/v) sucrose and 5.8 g/L agar (pH 5.8), as described by Ruhlman (Ruhlman, 2014). Tissue culture conditions were 23  °C at a photon flux of ~40 μmol photons m^−2^ s^−1^ (16 h light, 8 h dark). Once large enough, plants were transferred to soil, covered with a transparent cover for 4–5 days to allow for humidity adaptation, and grown in controlled environmental conditions under a diurnal cycle of 16 h light at 23 °C, 50% humidity, and 8 h darkness at 20 °C, 65% humidity. *C. reinhardtii* wild-type strain cc1690 was provided by the Chlamydomonas Culture Collection (University of Minnesota). Strains were grown on 2% (w/v) agar Tris acetate phosphate (Gorman and Levine, 1965) (TAP) plates at 23 °C at a photon flux of ~25 μmol photons m^−2^ s^−1^ (16 h light, 8 h dark). Seed tests were carried out on either MS, MS with spectinomycin (500 mg/L for tobacco, 50 mg/L for lettuce), or MS with spectinomycin and streptomycin (both 500 mg/L for tobacco, both 50 mg/L for lettuce).

### 2.2 Vector design and assembly

All novel plasmids constructed in this work were assembled using Golden Gate assembly (Engler et al., 2008). Codon-optimised myoglobin sequences of *Bos taurus* (*Bt Mb*) and *Sus scrofa* (*Ss Mb*) for tobacco and lettuce chloroplast were based on amino acid sequences retrieved from UniProt (accession numbers P02192 and P02189, respectively). Parental and novel plasmids are listed in the supporting information (**Supplementary Table 1**).

The myoglobin tobacco vector was constructed from tobacco chloroplast expression vector pKM014, provided by Kyomei Ltd. To construct pKM014, the codon-optimised *Bt Mb* gene (**Supplementary Table 2**) for tobacco chloroplast expression was first inserted into the *E. coli* expression vector pBSU0 (Neupert et al., 2008), replacing the *GFP* gene at NcoI and HindIII sites, to generate pKM013. The use of U0 as a 5′ UTR for the chloroplast is novel; 5′ UTRs used for *E. coli* have previously performed well for chloroplast transformation (Ruiz et al., 2011; Wang et al., 2023). The resulting expression cassette was excised as a SacI/HindIII fragment and ligated into the plastid transformation vector pKP9 (Zhou et al., 2008), yielding pKM014. The pKM014 backbone was amplified by PCR using Q5 Polymerase (New England Biolabs) with AG122 and AG123 (**Supplementary Table 3**). The porcine *Mb* gene sequence was optimised for tobacco chloroplast expression using OPTIMIZER (Puigbò et al., 2007) with the Kazusa database (Nakamura et al., 2000) (**Supplementary Table 2**). The generated sequence was commercially synthesised (Integrated DNA Technologies) with flanking PaqCI sites and assembled in the pKM014 backbone using PaqCI-mediated Golden Gate cloning to produce vector pAG108.

The myoglobin lettuce vector was constructed from lettuce chloroplast expression vector pKM058, provided by Kyomei Ltd. To construct pKM058, a modified version of pKP9, designated pKM023, was created by replacing the tobacco *trnfM–trnG* region with homologous lettuce sequences from the lettuce plastid genome (NC_007578). To enable Golden Gate cloning, undesired Type IIS restriction sites were removed via point mutations, and the BpiI-BsaI cassette from pStA212 (Taylor et al., 2019) was inserted between the HindIII and SacI sites. The entire pKM023 vector was synthesised by GenScript. The codon-optimised bovine *Mb* gene for lettuce chloroplasts was synthesised and cloned into pBSU0 to generate gKM011. Its expression cassette was then inserted into pKM023 between HindIII and SacI sites to construct pKM058. The pKM058 backbone was amplified by PCR using Q5 Polymerase with AG156 and AG157 (**Supplementary Table 3**). The porcine *Mb* gene sequence was optimised for lettuce chloroplast expression using the same approach (**Supplementary Table 2**). The native *psbA* promoter and 5′ UTR from lettuce (1541–1671 from GenBank: AP007232.1) followed by the chloroplast codon-optimised porcine Mb coding sequence was commercially synthesised (Integrated DNA Technologies) with flanking PaqCI sites and assembled in the pKM058 backbone using PaqCI-mediated Golden Gate cloning to produce vector pAG115.

Porcine *Mb* was also codon optimised for plant nuclear expression (**Supplementary Table 2**) by the same method and commercially synthesised (Integrated DNA Technologies) with flanking BsaI restriction sites. Nuclear transformation vectors for tobacco were assembled by BsaI-mediated Golden Gate using the plasmids listed: pICH51266 (CaMV35S promoter) or pICSL12015 (*Arabidopsis thaliana* ubiquitin 10 promoter), pICSL60004, and pJK403 (provided by Dr. Jiorgos Kourelis, Imperial College London) with the commercially synthesised nuclear *Mb* gene. The *Chlamydomonas* chloroplast transformation vector pAG102 was assembled by BsaI-mediated Golden Gate using the plasmids listed: pAG101 (provided by Prof. Peter Nixon, Imperial College London), pSS117, pHJ198, pHJ205, and pHJ192 (provided by Prof. Saul Purton, UCL). To build the *E. coli* vector expressing His-tagged porcine Mb (pAG112), AG134 and AG135 (**Supplementary Table 3**) were used to amplify the porcine *Mb* gene from pAG108 with Q5 Polymerase, which was assembled into plasmid pHT463 (provided by Dr. Henry Taunt, Imperial College London) by SapI-mediated Golden Gate.

The final transformation vectors were transformed into competent DH5-alpha *E. coli* cells (New England Biolabs) and purified using the QIAfilter Plasmid Midi Kit (QIAGEN) according to manufacturer instructions. Whole plasmid sequencing (Full Circle Labs, Ltd.) was performed for sequence confirmation.

### 2.3 Biolistic transformation

Leaves from 6-week-old tobacco and 3-week-old lettuce were used for biolistic transformation, as described previously (Maliga et al., 2021). Briefly, tobacco (1,100 psi, 9 cm distance, abaxial side up) and lettuce (1,200 psi, 9 cm distance, adaxial side up) leaves were bombarded with 0.6-μm gold microcarriers using a PDS-1000/He Biolistic Particle Delivery System (Bio-Rad) on regenerative media (MS supplemented with 3% (w/v) sucrose, 1 mg/L 6-benzylaminopurine (BAP), 1 mg/L thiamine, 0.1 mg/L 1-naphthaleneacetic acid (NAA), 0.1 g/L *myo*-inositol, and 5.8 g/L agar, pH 5.8, for tobacco; MS supplemented with 3% (w/v) sucrose, 0.2 mg/L BAP, 0.1 mg/L NAA, 0.5 mg/L polyvinylpyrrolidone, 0.1 g/L *myo*-inositol, and 5.8 g/L agar, pH 5.8, for lettuce). After 2 days of incubation in the dark at 23 °C, the leaves were cut into explants and placed on regenerative media supplemented with selection (500 mg/L spectinomycin for tobacco; 50 mg/L spectinomycin for lettuce) until shoots appeared. Shoots were regenerated several times on selection media and then transferred to MS supplemented with the appropriate selection. For *C. reinhardtii*, cells were inoculated into 50 mL liquid TAP medium and cultured over several days at 23 °C with shaking (140 rpm), until the optical density at 750 nm (OD_750_) reached 0.4-0.7. 40 mL of culture was harvested by centrifugation (4,000 × *g*, 20 min, 22 °C) and cells were concentrated 20-fold. 200 µL of cells was spread onto 2% (w/v) agar TAP plates supplemented with 150 mg/L spectinomycin. The cells were bombarded as described previously (Taunt et al., 2023) and incubated at 23 °C at a photon flux of ~5 μmol photons m^−2^ s^−1^ (16 h light, 8 h dark) for 24 h followed by ~20 μmol photons m^−2^ s^−1^ (16 h light, 8 h dark) until visible colonies formed. These colonies were restreaked under increasing selection pressure: TAP supplemented with 300 mg/L spectinomycin and subsequently TAP with 500 mg/L spectinomycin and 50 mg/L streptomycin.

### 2.4 Agrobacterium-mediated transformation

300–500 ng of plasmid DNA and 50 µL of electrocompetent *Agrobacterium tumefaciens* AGL0 cells (provided by Dr. Karen Sarkisyan, Imperial College London) were mixed; electroporation was performed by the MicroPulser Electroporator (Bio-Rad) on setting “Agr”. After adding 400 µL LB (Sezonov et al., 2007), transformed cells were incubated for 1 h at 28 °C with shaking (200 rpm) and then plated onto LB supplemented with 50 mg/L kanamycin, 50 mg/L streptomycin, and 25 mg/L rifampicin. The plates were incubated at 28 °C for 2–3 days until colonies appeared. Colonies were inoculated into 20 mL of liquid MS and incubated at 28 °C with shaking (200 rpm) until OD_600_ reached 0.6.

Leaves from 10-day-old tobacco seedlings were cut in half and incubated in the *Agrobacterium* culture for 20 min (Mitiouchkina et al., 2020). Explants were placed onto regenerative media (as for biolistics) in the dark at 23 °C for 2 days and then transferred to regenerative media supplemented with 75 mg/L kanamycin and 500 mg/L cefotaxime. Shoots were then transferred to MS supplemented with 100 mg/L kanamycin and 300 mg/L cefotaxime.

### 2.5 Genotyping

#### 2.5.1 Genotyping by Southern blot

Total DNA was extracted from leaf tissue using the GeneJET Plant Genomic DNA Purification Kit (Thermo Scientific). DNA samples (3 μg for lettuce, 5 μg for tobacco) were digested with the restriction enzyme BglII (New England Biolabs) and separated by gel electrophoresis in a 0.8% agarose/Tris-acetate-EDTA (TAE) gel. Denaturation, neutralisation, and capillary transfer to a positively charged nylon membrane (Roche) were performed as described in previous literature (Southern blotting: Capillary transfer of DNA to membranes, 2004). The 541-bp tobacco probe template from the *psaB* gene was amplified from the tobacco genomic DNA by PCR using AG208 and AG209 (**Supplementary Table 3**) with Q5 Polymerase and purified by running the PCR product on a 1% agarose/TAE gel and using the GeneJET Gel Extraction Kit (Thermo Scientific) according to the manufacturer’s instructions. The 542-bp lettuce probe template from the *psaB* gene was amplified from the lettuce genomic DNA using the same approach with AG145 and AG212 (**Supplementary Table 3**) and purified using the GeneJET PCR Purification Kit (Thermo Scientific) according to the manufacturer’s instructions. Probe synthesis, hybridisation, and detection were performed using the DIG-High Prime DNA Labelling and Detection Starter Kit II (Roche) according to the manufacturer’s instructions using positively charged nylon membranes (Roche).

#### 2.5.2 Genotyping by sequencing

Total DNA was extracted from young leaves of tobacco and lettuce plants and sequenced by Wuhan Benagen Technology Company Ltd. Library preparation followed the manufacturer’s short−read protocol: genomic DNA was sheared to ~350 bp with ultrasonic fragmentation, end−repaired, A−tailed, ligated to Illumina adapters, purified, and PCR−enriched. Libraries were quantified with a Qubit 2.0 fluorometer, sized on an Agilent 2100 Bioanalyzer, and validated by qPCR. Paired−end sequencing (2 × 150 bp) was performed on an Illumina NovaSeq 6000 S4 flow−cell, generating >10-Gb clean data per sample. Raw reads were quality−trimmed (Phred ≥30) and aligned in Geneious Prime v.2023.2.1 (Biomatters Ltd.) to the reference chloroplast genomes of Nicotiana tabacum (NC_001879) and Lactuca sativa (NC_007578) using default settings. Reads-to-reference pairwise identity was calculated as the mean across all mapped reads of (number of matched bases) ÷ (total aligned positions, including mismatches and gap columns) × 100%. Consensus sequences were generated using a default threshold of 60% (bases matching at least 60% of total adjusted chromatogram quality). Consensus and variant sequences were visualised and counted in Geneious Prime.

#### 2.5.3 Genotyping by PCR

Genomic DNA from *Chlamydomonas* was extracted by diluting cells in 50 µL of 5% (w/v) Chelex 100 resin (Bio-Rad). The sample was then vortexed, boiled for 10 min at 95 °C, and centrifuged (17,000 × *g*, 1 min, 22 °C). 1 µL of supernatant was used as template per 20-µL PCR reaction alongside HT492, HT493, and HT494 (**Supplementary Table 3**) with Phire Plant Direct PCR Master Mix (Thermo Scientific) according to the manufacturer’s instructions; products were run on a 1% agarose/TAE gel.

### 2.6 SDS-PAGE and Western blotting

Leaf tissue (100 mg) from one plant per line grown in controlled environmental conditions was lysed using the TissueLyser II (QIAGEN) under liquid nitrogen. 200 µL of native protein extraction buffer (PBS (137 mM NaCl, 2.7 mM KCl, 10 mM Na_2_HPO_4_, 1.8 mM KH_2_PO_4_), 5 mM ascorbic acid, 2% (w/v) polyvinylpyrrolidone, 1 cOmplete™ EDTA-free Protease Inhibitor Cocktail tablet (Roche)/50 mL) was then added to the tissue. The sample was thawed on ice and further homogenised by vortexing at 4 °C for 1 min. The lysate was then centrifuged (15,000 × *g*, 10 min, 4 °C).

Cultures of *C. reinhardtii* strains (2 L) were grown in liquid TAP until OD_750_ reached 1. Cells were centrifuged (4,000 × *g*, 20 min, 22 °C) and resuspended in 50 mL buffer A (200 mM NaCl, 50 mM Tris–HCl, pH 8.5 buffer, 1 cOmplete™ Protease Inhibitor Cocktail (Roche)/50 mL). The resuspended cells were passed through the precooled Continuous Flow Cell Disruptor (Constant Systems) at 16 kpsi. The lysate was quantified by Bradford assay (Sigma) using bovine serum albumin (BSA) as a standard. The lysate was then ultracentrifuged using the Type 45 Ti Fixed-Angle Rotor (Optima XPN Ultracentrifuge, Beckman Coulter, 35–000 rpm, 40 min, 4 °C). The insoluble fraction was solubilised in lysis buffer (0.1 M NaOH, 0.05 M EDTA, 2% SDS (w/v)) using a paintbrush and quantified using the Pierce™ BCA Protein Assay Kit (Thermo Scientific) with BSA as a standard.

*Chlamydomonas* samples were quantified using a bovine Mb standard (Worthington Biochemical Corporation), and plant samples were quantified using a porcine Mb standard previously purified from *E. coli.* Protein samples were separated by electrophoresis in 10%–20% Novex™ Tris-Tricine Mini Protein Gels or 4%–20% Novex™ Tris-Glycine Mini Protein Gels (Thermo Scientific). Two gels were run in parallel: after running, one gel was stained overnight in Quick Coomassie Stain (Protein Ark). The other gel was transferred onto a nitrocellulose membrane. Mb was detected with 1:2,000 Rabbit Anti-Sheep Mb primary antibody (ab231725; Abcam) followed by 1:10,000 secondary Goat Anti-Rabbit IgG HRP Affinity Purified antibody (HAF008; Bio-Techne). SuperSignal West Pico PLUS Chemiluminescent Substrate (Thermo Scientific) was applied to the membrane for visualisation. Densitometry was carried out using FIJI (Schindelin et al., 2012). The membrane was stained using Ponceau Staining Solution (5% (v/v) glacial acetic acid, 0.1% (w/v) Ponceau S) to visualise the loading control.

### 2.7 Protein purification

#### 2.7.1 Mb purification from tobacco

Mb from tobacco was purified by crushing 20 g of the mature leaves (from several 7-week-old plants grown in controlled environmental conditions) into a fine powder with a mortar and pestle in liquid nitrogen. The crushed leaves were then resuspended in 50 mL of buffer B (1mM DTT, 50 mM Tris–HCl, pH 8.5 supplemented with 1 cOmplete™ EDTA-free Protease Inhibitor Cocktail tablet (Roche)/50 mL). The lysate was ultracentrifuged using the Type 45 Ti Fixed-Angle Rotor (Optima XPN Ultracentrifuge, Beckman Coulter, 20–000 rpm, 20 min, 4 °C). The supernatant was filtered through a 0.2-μm syringe filter (Sartorius), and the protein concentration was measured by Bradford assay (Sigma) using BSA as a standard.

For anion exchange chromatography, the Mono Q™ 5/50 GL column (Cytiva) was used, operated by the ÄKTA pure™ chromatography system (Cytiva) at 4 °C. The column was equilibrated with buffer C (50 mM Tris–HCl, pH 8.5). Once the sample was loaded on the system, the column was washed with 10 column volumes of buffer C. An elution gradient of 0–100 mM NaCl over 40 column volumes was applied to the column, as 0.5 mL fractions were collected. The flowthrough was concentrated with 3K molecular weight cut-off (MWCO) MilliporeSigma™ Amicon™ Ultra-15 Centrifugal Filter Units (Sigma) to a final volume of 1 mL and buffer exchanged into buffer A. Mb was purified by size exclusion chromatography using the HiLoad 16/600 Superdex 75 pg column (Cytiva), operated by the ÄKTA pure™ chromatography system (Cytiva) at 4 °C. The column was equilibrated with buffer A. Mb eluted from the column within one column volume of buffer A, as determined by the 280- and 490-nm absorbance readings. All purified proteins were stored by adding 20% glycerol (v/v) final volume, aliquoted, and flash frozen in liquid nitrogen prior to storage at −80 °C.

#### 2.7.2 Mb purification from *E. coli*

For *E. coli*, KRX cells (Promega) were transformed with pAG112 according to the manufacturer’s instructions. Colonies were inoculated into 10–40 mL liquid LB overnight, supplemented with the appropriate antibiotic, and then scaled up to 1-4 L in Terrific Broth (TB) and incubated at 37 °C (200 rpm) until OD_600_ reached 0.8–1. Protein production was induced by adding 1 mL of 1 M isopropyl β-D-thiogalactopyranoside (IPTG) and 5 mL of 1.22 M L-rhamnose per litre of culture and incubating overnight at 18 °C. The cells were harvested by centrifugation (4,000 × *g*, 30 min, 22 °C), resuspended in 50 mL of buffer A, and then passed twice through the pre-cooled Continuous Flow Cell Disruptor at 32 kpsi. The lysate was ultracentrifuged using the Type 45 Ti Fixed-Angle Rotor (Optima XPN Ultracentrifuge, Beckman Coulter, 30,000 rpm, 40 min, 4 °C). The ultracentrifugation supernatant was added to 4 mL of equilibrated Super Ni-NTA agarose resin (Protein Ark) and incubated overnight at 4 °C with rotation. The lysate–resin mixture was then centrifuged (1,000 × *g*, 5 min, 4 °C). 10 mL of wash (200 mM NaCl, 50 mM Tris–HCl, 10 mM imidazole, pH 8.5) was added, shaken, and centrifuged (1,000 × *g*, 5 min, 4 °C). This step was repeated twice more.

This process was repeated twice with 10 mL of elution solution 1 (200 mM NaCl, 50 mM Tris–HCl, 300 mM imidazole, pH 8.5) and once with 10 mL of elution solution 3 (200 mM NaCl, 50 mM Tris–HCl, 500 mM imidazole, pH 8.5). The elution fractions were pooled and concentrated (MWCO 3K) down to 10 mL. At this stage, the protein concentration was measured by Bradford assay (Sigma) using BSA as a standard. 60 units of thrombin (MP Biomedicals) was added per mg of protein. This was incubated at 37 °C overnight with shaking (200 rpm). To capture remaining His-tagged proteins, the digestion mixture was incubated with equilibrated Ni-NTA agarose resin for 1 h at 4 °C with rotation. The lysate–resin mixture was then centrifuged (1,000 × *g*, 5 min, 4 °C). The supernatant was concentrated (MWCO 3K) to 1 mL.

Mb was purified by size exclusion chromatography using the HiLoad 16/600 Superdex 75 pg column (Cytiva) and operated by the ÄKTA pure™ chromatography system (Cytiva) at 4 °C. The column was equilibrated with buffer A. Mb eluted from the column within one column volume of buffer A, as determined by the 280- and 490-nm absorbance readings.

### 2.8 Protein analysis

Spectrophotometry was measured using the Cary 60 UV-Vis Spectrophotometer (Agilent Technologies) over 250–700 nm in a 1-cm quartz cuvette. Mb was analysed by LC-MS using the 1290 Infinity II UHPLC (Agilent Technologies) coupled with AdvanceBio 6545XT LC/QTOF (Agilent Technologies). The protein sample was first buffer exchanged into LC-MS-grade H_2_O at 4 °C (MWCO 3K). The gradient (0.1% (v/v) formic acid in water as A and 0.1% (v/v) formic acid in acetonitrile as B) was 0%-20% B 0–1 min, 20%-100% B 1–3 min, 100% B 3–4 min, and then 0% B 4–5 min. Data were acquired using Agilent MassHunter for LC/QTOF 12.0 and deconvoluted by Agilent BioConfirm 12.0. The circular dichroism (CD) spectra were measured using the Chirascan V100 CD spectrometer (Applied Photophysics). The masses were analysed by searching for masses using the expected amino acid sequence, with and without the N-terminal methionine.

A sample of ~0.1 mg/mL in 100 mM phosphate buffer (75 mM Na_2_HPO_4_, 25 mM NaH_2_PO_4_, pH 7) was measured in a 1-mm quartz cuvette. The average of three spectrum scans was calculated. The pyridine hemochromagen assay was carried out as described previously (Barr and Guo, 2015); calculations were done using the reduced pyridine hemochromagen spectrum with its extinction coefficient of 34.7 mM^−1^ cm^−1^ at 557 nm (Paul et al., 1953) for heme *b.*

### 2.9 Plant physiology analyses

All plant physiology analyses were measured using the fifth leaf from the top of 8-week-old tobacco plants grown in controlled environmental conditions; the averages and standard deviations were plotted from three biological replicates (except dry weight). Statistical significance was calculated Welch’s t-test (p < 0.05) unless otherwise stated. Photosynthetic parameters were measured using the DUAL-PAM-100 instrument (Heinz Walz GmbH) at 22 °C when the first flower of the plant was about to open. After 30 min of dark adaptation, the maximum quantum efficiency of photosystem II (PSII) in the dark-adapted state (F_v_/F_m_) was measured. Light response curves were measured for parameters of ETR(II), Y(II), and Y(NPQ). The measuring times at each actinic light intensity were 150 s under light-limited and 60 s under light-saturated conditions. Statistical significance was calculated using two-way repeated-measures ANOVA (genotype × light intensity, p < 0.05). Chlorophyll concentrations were determined as described previously (Espinas et al., 2012) using the Cary 60 UV-Vis Spectrophotometer (Agilent Technologies) at 663 and 645 nm; chlorophyll *a* and *b* concentrations were calculated as described previously (Lichtenthaler and Wellburn, 1983).

Heme measurement was adapted from Espinas et al. (2012); the leaf sample (100 mg) was powdered under liquid nitrogen and extracted in 300 μL of 80% (v/v) acetone containing 20% (v/v) 1.6 M HCl. After 5 min of incubation at room temperature, the sample was centrifuged (17,000 × *g*, 10 min, 22 °C). Supernatants were diluted 100-fold with ddH_2_O. 10 μL of the diluted samples was mixed with 40 μL of apo-HRP (BBI solutions; 250 nM final concentration in 100 mM Tris–HCl, pH 8.4) and incubated at 30 min on a shaker (50 rpm). 50 μL of Thermo Scientific SuperSignal West Pico PLUS Chemiluminescent Substrate (Thermo Scientific) was added, and after 5 min of incubation, the chemiluminescence signal was quantified using a white 96-well plate (Greiner Bio-One) and the CLARIOstar Plus (BMG Labtech) microplate reader with integration time 0.5 s. The values were compared with known bovine hemin (Sigma) concentrations; 1 mM stock was prepared in DMSO and then diluted with ddH_2_O.

The dry weight of tobacco and lettuce leaves was determined by weighing leaf samples and incubating them in a 65 °C oven, re-weighing every 2–3 h until constant mass. Averages and standard deviations were calculated from five biological replicates. Plant pictures were taken with an iPhone 16 (Apple).

## 3 Results

### 3.1 Generation of transplastomic tobacco, lettuce, and *Chlamydomonas* Mb-expressing lines

**Figure 1:**
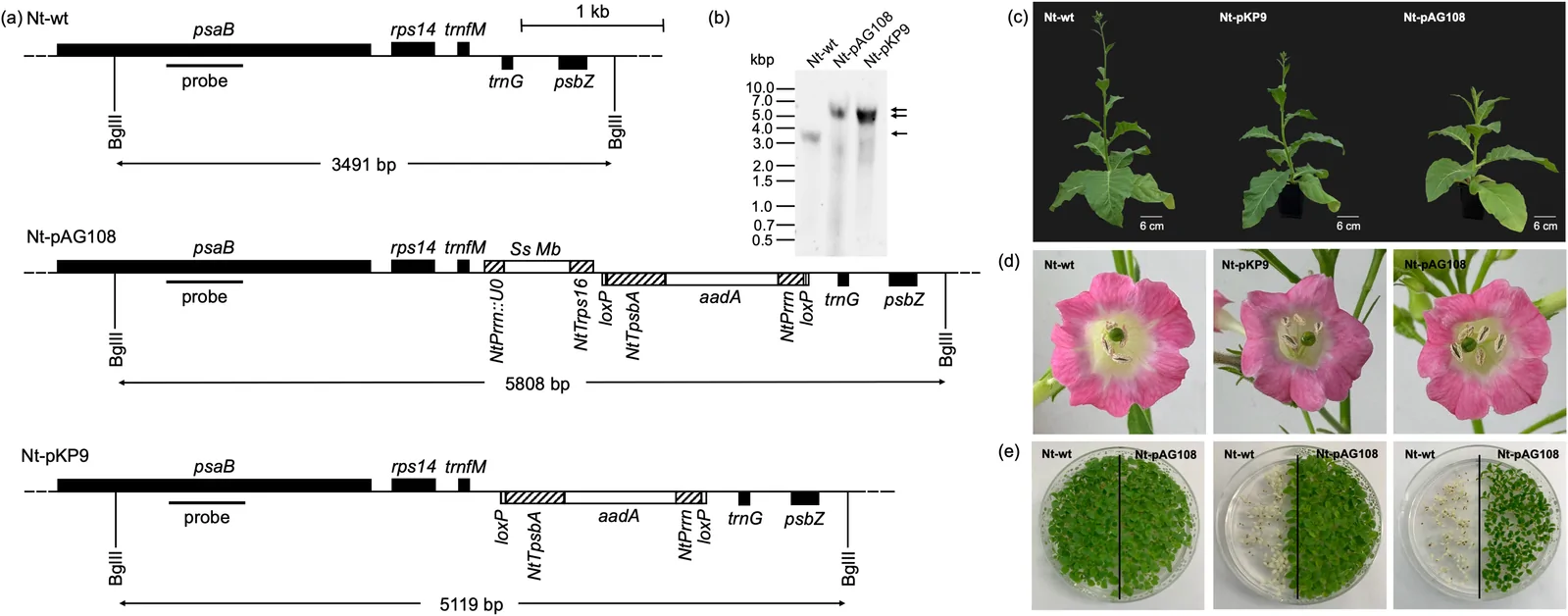
Generation of tobacco plastid transformants with vectors pKP9 and pAG108. **(a)** Map of the target region within the tobacco plastid genome and the design of vectors pKP9 and pAG108 used for tobacco plastid transformation. In both vectors, the transgenes are targeted to the intergenic region between *trnfM* and *trnG.* The selectable marker gene *aadA* is driven by the ribosomal RNA operon promoter (NtPrrn) followed by the 3′ UTR from the plastid *psbA* gene (NtTpsbA), with flanking *loxP* sites for downstream marker removal (Lutz et al., 2006). The porcine myoglobin (Mb) gene (*Ss Mb*) is driven by the NtPrrn promoter followed by a synthetic U0 5′ UTR (NtPrrn::U0) followed by the 3′ UTR from the small ribosomal subunit (NtTrps16). **(b)** Southern blot of total genomic DNA of wild-type (Nt-wt), empty vector control (Nt-pKP9), and Mb-expressing mutant (Nt-pAG108) after digestion by BglII and hybridisation using a DNA probe shown in **(a)**. **(c)** Growth phenotype of Nt-wt (left), Nt-pKP9 (middle), and Nt-pAG108 (right) 40 days after transfer to soil. **(d)** Flowers of Nt-wt (left), Nt-KP9 (middle), and Nt-pAG108 (right) 60 days after transfer to soil. **(e)** Inheritance assay of wild-type and transplastomic tobacco plants. Seeds from Nt-wt and Nt-pAG108 plants were germinated on medium lacking selection (left), containing spectinomycin (middle) or spectinomycin and streptomycin (right).

**Figure 2:**
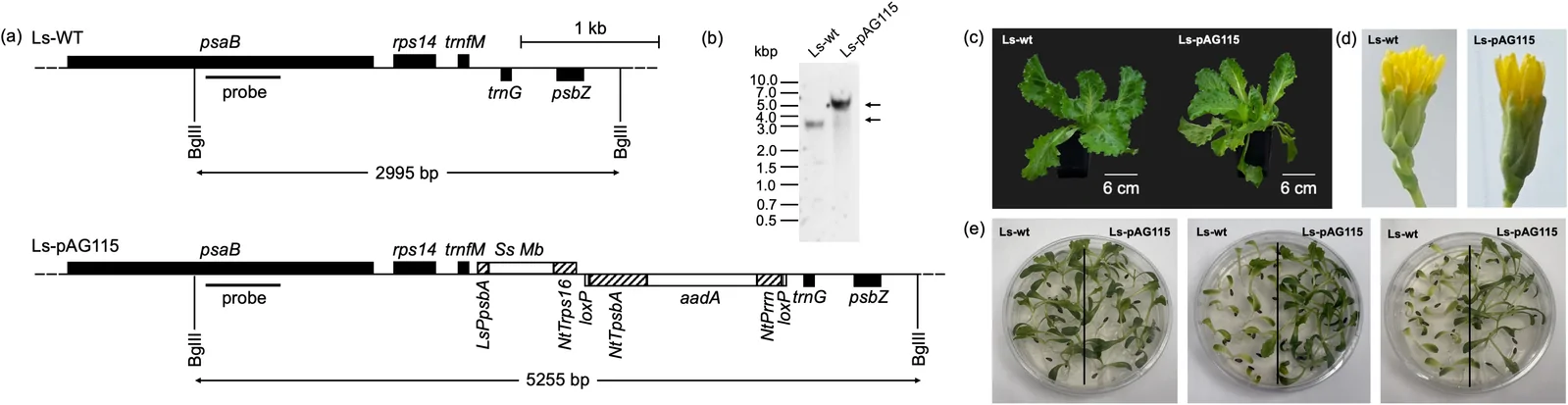
Generation of lettuce plastid transformants with vector pAG115. **(a)** Map of the target region within the lettuce plastid genome and the design of vector pAG115 used for lettuce plastid transformation. The transgenes are targeted to the intergenic region between *trnfM* and *trnG.* The selectable marker gene *aadA* is driven by the chimeric tobacco ribosomal RNA operon promoter (NtPrrn) followed by the 3′ UTR from the tobacco plastid *psbA* gene (NtTpsbA), with flanking *loxP* sites for downstream marker removal. The porcine myoglobin (Mb) gene (*Ss Mb*) is driven by the endogenous lettuce *psbA* promoter and 5′ UTR (LsPpsbA) followed by the 3′ UTR from the tobacco small ribosomal subunit (NtTrps16). **(b)** Southern blot of total genomic DNA of wild-type (Ls-wt) and Mb-expressing mutant (Ls-pAG115) after digestion by BglII and hybridisation using a DNA probe shown in **(a)**. **(c)** Growth phenotype of Ls-wt (left) and Ls-pAG115 (right) 65 days after transfer to soil. **(d)** Flowers of Ls-wt (left) and Ls-pAG115 (right) 100 days after transfer to soil. **(e)** Inheritance assay of wild-type and transplastomic lettuce plants. Seeds from Ls-wt and Ls-pAG115 plants were germinated on medium lacking selection (left), containing spectinomycin (middle) or spectinomycin and streptomycin (right).

Vectors for myoglobin expression were designed for each organism. For tobacco, the pAG108 construct was based on pKP9 (Oey et al., 2009), which is used as an empty vector control inserting into the *psaB* locus (**Figure 1a**). The ribosomal RNA operon promoter from tobacco (Prrn) was chosen as a strong promoter (Verhounig et al., 2010) and bacterial U0 sequence as the synthetic 5′ UTR (Neupert et al., 2008) for porcine Mb expression (**Figure 1a**). Equivalent endogenous *psaB* insertion sequences were used for the lettuce vector pAG115, and the *psbA* promoter and 5′ UTR were used to drive porcine Mb expression (Ruhlman et al., 2010) (**Figure 2a**). For the *Chlamydomonas* pAG102 vector, the 16S rRNA promoter and *psaA* 5′ UTR were used for bovine Mb expression (Taunt et al., 2023), and insertion was targeted to the *psbA* locus (**Supplementary Figure 1a**).

The constructs were introduced into plastids by biolistic bombardment (Ruhlman, 2014; Maliga et al., 2021). For *Chlamydomonas*, several colonies were obtained, whereas for both tobacco and lettuce, only one mutant plant line was obtained. Antibiotic-resistant lines were subjected to several rounds of selective regeneration. For lettuce and tobacco, Southern blotting analysis (**Figures 1b**, **2b**) and genome sequencing (**Supplementary Figures 2a-d**) confirmed successful transformation and that the mutant plants were homoplasmic. While previous studies examine homoplasmy using only Southern blotting (Ruhlman et al., 2010; Fatima et al., 2025), here genome sequencing was used as a complementary tool to achieve higher resolution without relying on the availability of restriction sites. Similar approaches are already well-established for mitochondrial DNA (Mok et al., 2024). For *Chlamydomonas*, homoplasmy was confirmed by PCR analysis (**Supplementary Figure 1b**).

Tobacco and lettuce transformants had normal morphology (**Figures 1c**, **2c**) and were able to produce fertile flowers (**Figures 1d**, **2d**) and functional seeds which survived when sowed on selection, demonstrating the stable nature of the transformation (**Figures 1e**, **2e**). Tobacco and lettuce seed assays revealed that the progenies of the transplastomic lines showed no segregation on spectinomycin and streptomycin-containing media as expected for chloroplast transformants; of the 200 seeds sown, Nt-pAG108 seeds had a 93 ± 4.4% germination efficiency and a 0% bleaching efficiency as opposed to Nt-wt seeds which had a 96 ± 1.6% germination efficiency and a 100% bleaching efficiency (**Supplementary Figure 3a**). Ls-pAG115 seeds had an 81.5 ± 2.0% germination efficiency and a 0% bleaching efficiency, as opposed to Ls-wt seeds which had an 85.5 ± 1.5% germination efficiency and an 100% bleaching efficiency (**Supplementary Figure 3b**). When grown in soil under controlled conditions, the transplastomic tobacco lines Nt-KP9 and Nt-pAG108 show a growth retardation phenotype compared with Nt-wt, but no growth stunting phenotype (**Supplementary Figure 4**).

### 3.2 Confirmation and quantification of Mb accumulation in transplastomic lines

**Figure 3:**
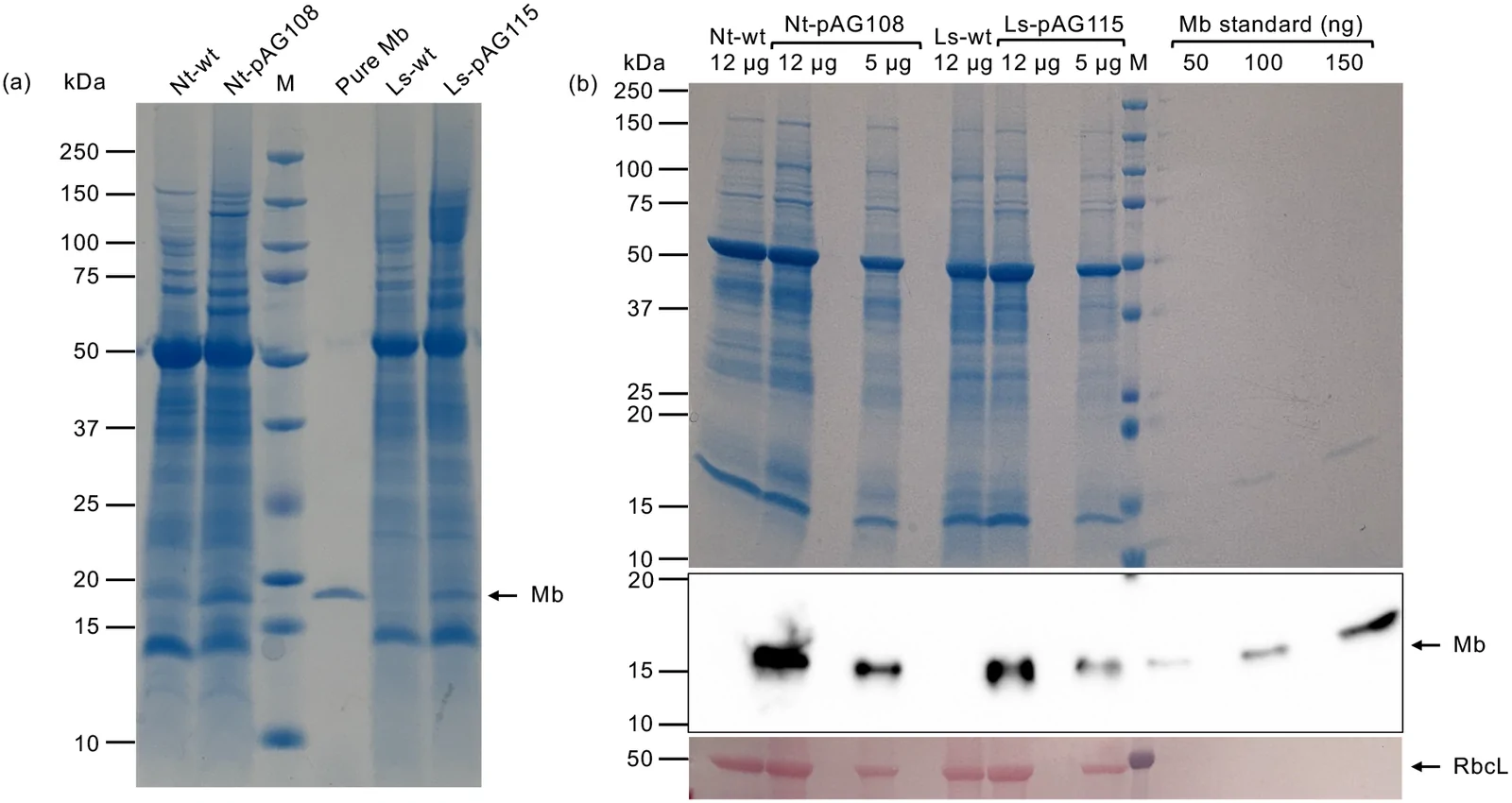
Mb quantification in tobacco and lettuce transformants. **(a)** SDS-PAGE of total soluble protein from wild-type plants and Mb-expressing mutants. Pure Mb was included as a size control. **(b)** Immunoblot analysis of tobacco and lettuce wild-type plants and Mb-expressing mutants. Immunoblot of total soluble proteins (middle) using an α-Mb antibody confirms Mb expression and Mb standards (50, 100, and 150 ng) allow for quantification of Mb expression by densitometry in tobacco and lettuce as a proportion of TSP. The replicate stained SDS-PAGE gel (top) and Ponceau S staining of the membrane (bottom) show even loading.

Analysis of the total soluble protein (TSP) fraction from leaves by SDS-PAGE and Coomassie Blue staining revealed an additional band at 17 kDa in both tobacco and lettuce transplastomic plants (**Figure 3a**) that was also detected by immunoblotting (**Figure 3b**). Using the standard curve from known Mb standards (**Supplementary Figure 5**), the porcine Mb protein accumulation was estimated by densitometry (Ruhlman et al., 2007; Davoodi-Semiromi et al., 2010; Gray et al., 2011) and found to be around 2.8% of TSP in tobacco and around 1.5% of TSP in lettuce. To demonstrate reproducibility, porcine Mb protein was quantified by densitometry in the same Nt-pAG108 tobacco transformant from another immunoblot (**Supplementary Figure 6**), with an estimated quantification of 2.6% TSP.

As a comparison, nuclear transformation of tobacco using the cauliflower mosaic virus 35S promoter (Schnurr and Guerra, 2000) and the *Arabidopsis thaliana* ubiquitin 10 promoter (Grefen et al., 2010) was also performed (**Supplementary Figures 7a**, **8a**). Of the 37 nuclear transformant plants analysed, porcine Mb accumulation was consistently lower (minimum 3-fold) in the leaves of nuclear transformed plants compared with transplastomic plants (**Supplementary Figures 7b**, **8b**). Mb accumulation in *C. reinhardtii* was estimated to be <0.25% TSP, much lower than both tobacco and lettuce (**Supplementary Figure 1c**). A doublet band is observed in *C. reinhardtii*, likely due to a degradation product of Mb (**Supplementary Figure 1c**).

### 3.3 Porcine Mb purification and analysis

**Figure 4:**
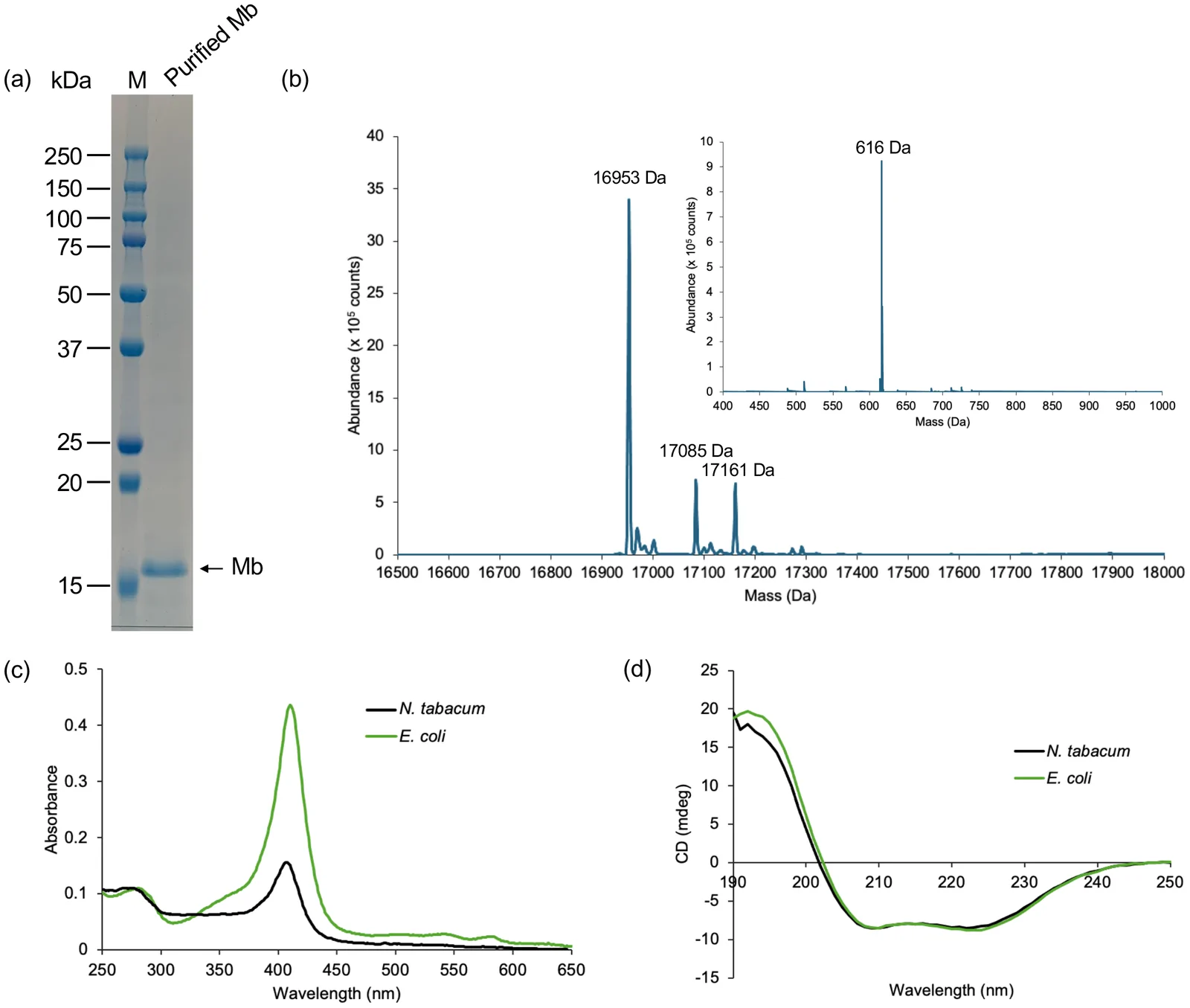
Purification and analysis of porcine Mb from transplastomic tobacco leaves. **(a)** Confirmation of Mb purification from tobacco leaves. Purified Mb was analysed by SDS-PAGE to confirm purity and approximate size of the purified product. **(b)** Mass spectrometric analysis of purified Mb. Purified porcine Mb from transplastomic tobacco leaves was analysed by LC-MS. Heme from the same sample was also analysed. **(c)** Absorbance of purified Mb. Absorbance of tobacco-expressed porcine Mb was measured from 250 to 650 nm (black); *E. coli-*expressed Mb was included for comparison (green). **(d)** Circular dichroism of purified Mb. The circular dichroism of tobacco-expressed porcine Mb was measured from 190 to 250 nm (black); *E. coli-*expressed Mb was included for comparison (green).

Due to its higher protein accumulation, porcine Mb was purified from tobacco leaves by anion-exchange chromatography followed by size-exclusion chromatography to yield a highly pure sample (**Figure 4a**). LC-MS analysis of the purified porcine Mb protein (full-length expected size of 17,085 Da) showed a single prominent peak with a mass of 16,953 Da, which corresponds exactly to the removal of the initiator methionine (**Figure 4b**), a common post-translational modification (PTM) in the chloroplast (Apel et al., 2010; Gisby et al., 2011). There are also two secondary peaks, at 17,085 Da and 17,161 Da. The 17,085-Da peak likely relates to Mb with its initiator methionine (predicted mass 17,085 Da), whereas the 17,161 Da peak may be due to a contaminant protein or PTMs. From the LC-MS spectrum, there is very little evidence of oxidative damage of the protein, which could be a risk due to the oxidative environment of the chloroplast (Foyer and Hanke, 2022). LC-MS also confirms the presence of heme *b* in the sample, expected at 616 Da (Bowman and Bren, 2008); no other types of heme were observed. An absorbance band was observed at 409 nm (**Figure 4c**), which is indicative of the presence of metmyoglobin (where Mb heme iron is oxidised to Fe^3+^), as opposed to other forms of myoglobin such as oxymyoglobin where the peak is observed at 418–420 nm (Millar et al., 1996). However, the ratio between the 409- and 280-nm peak was around 1.5, which is less than the ratio of *E. coli-*expressed Mb ratio of 4. This suggests that there is a significant portion of apo-Mb (Mb without heme bound) present in the sample.

To further investigate this, the pyridine hemochromatogen assay (Barr and Guo, 2015) was used to quantify how much heme was present in the sample (**Supplementary Figure 9**). By using an extinction coefficient of 34.7 mM^−1^ cm^−1^ at 557 nm (Paul et al., 1953) and relating this to the protein concentration determined by Bradford assay using BSA as a standard, the percentage of holo-Mb (Mb with heme bound) was estimated to be around 35%. By comparison, the percentage of heme-binding in *E. coli-*expressed Mb was 80%. The circular dichroism spectrum showed a typical alpha helix pattern as expected for proper folding of Mb (Almeida Ribeiro et al., 2003); the same pattern was observed for *E. coli-*expressed Mb (**Figure 4d**).

### 3.4 Physiology measurements of transplastomic Mb-expressing plants

**Figure 5:**
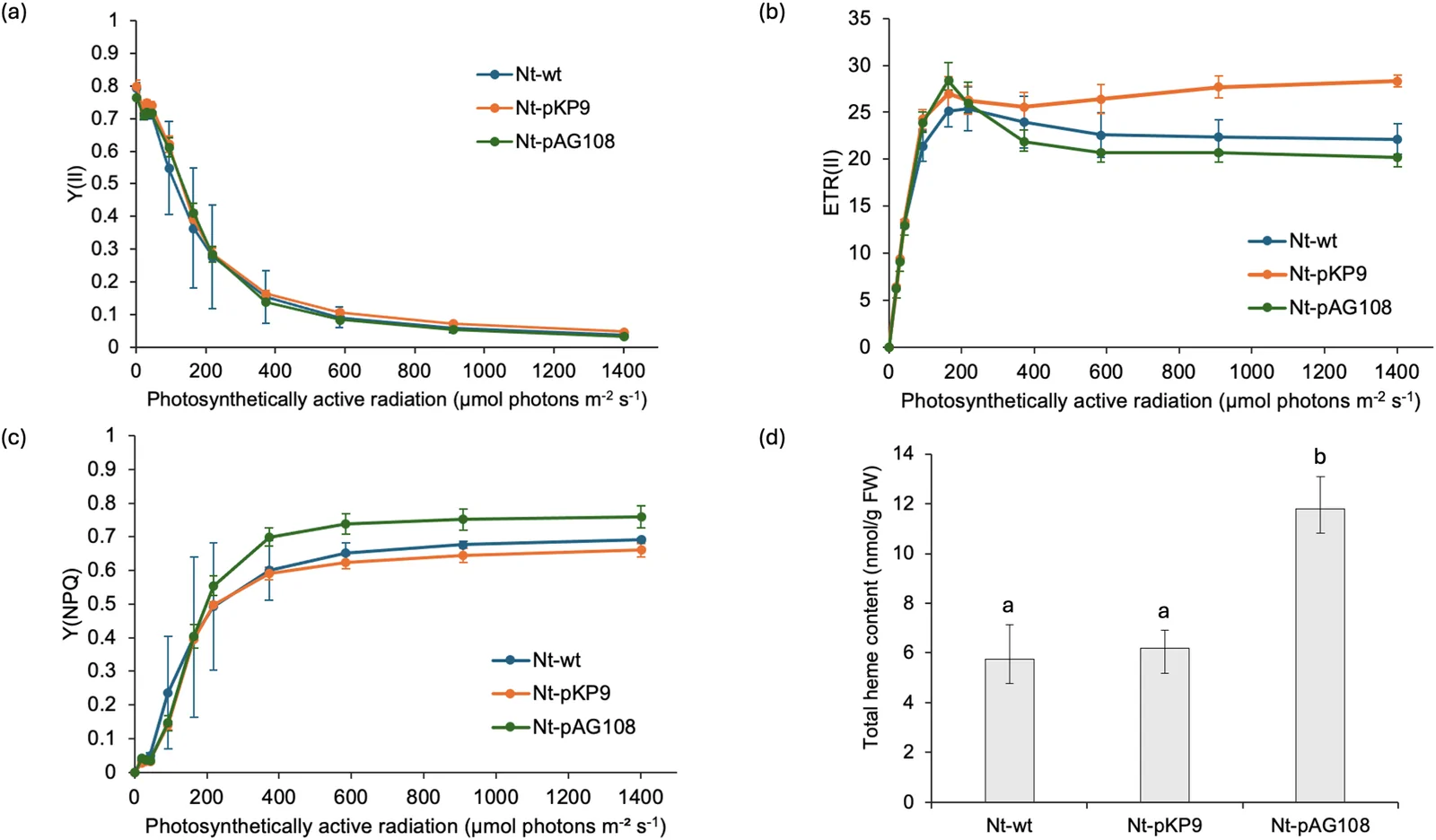
Photosynthetic and heme measurements in transplastomic tobacco leaves. **(a-c)** Photosynthetic parameters of wild-type and transplastomic tobacco. Effective quantum yield of photosystem II (Y(II)), electron transport rate through photosystem II (ETR(II)), and quantum yield of non-photochemical quenching (Y(NPQ)) are measured for Nt-wt (blue), Nt-pKP9 (orange), and Nt-pAG108 (green) as a function of increasing light intensity. Error bars represent standard deviations. **(d)** Total heme of wild-type and transplastomic tobacco. Total heme content is measured for Nt-wt, Nt-pKP9, and Nt-pAG108. Error bars represent standard deviations and letters represent groups that differ significantly based on Welch’s t-test (p < 0.05).

To study the impact of constitutive Mb production on the physiology of the transplastomic tobacco mutants, photosynthetic parameters were measured using a pulse-amplitude-modulation (PAM) fluorometer (**Figures 5a–c**). Of the parameters measured (effective quantum yield of photosystem II, Y(II); electron transport rate through photosystem II, ETR(II); quantum yield of non-photochemical quenching, Y(NPQ)), an insignificant difference was observed between Nt-wt, Nt-pKP9, and Nt-pAG108, suggesting that Mb expression did not significantly compromise photosynthetic processes. Total chlorophyll was also measured; Nt-pKP9 (1.98 ± 0.04 mg/g FW) and Nt-pAG108 (1.95 ± 0.05 mg/g FW) showed a significant increase compared with Nt-wt (1.55 ± 0.04 mg/g FW). Interestingly, whilst the chlorophyll *a* to chlorophyll *b* ratio appears equivalent between Nt-wt (3.59 ± 0.15) and Nt-pKP9 (3.56 ± 0.22), there was a slight decrease for Nt-pAG108 (3.18 ± 0.06). Total heme levels determined using the established apo-horseradish peroxidase assay were observed to be similar between Nt-wt and Nt-pKP9; however, Nt-pAG108 showed a twofold increase in total heme levels (**Figure 5d**).

## 4 Discussion

In this work, we have produced Mb in the chloroplast of higher plants. The porcine Mb purified from tobacco leaves shows similar physical properties to other mammalian Mb proteins in terms of size, folding, and heme binding. LC-MS reveals that the predominant Mb form lacks the initiator methionine, a common PTM for Mb that also occurs in mammals such as pigs (Rousseaux et al., 1976; Smerdon et al., 1990). It has been previously demonstrated that removal of the initiator methionine causes only minor changes to the global fold and little to no change to the heme pocket (Phillips et al., 1990). LC-MS also suggests there is little oxidative damage to the protein. Two other minor LC-MS peaks were observed, one relating to Mb with the initiator methionine retained and another unidentified peak, potentially relating to a contaminant protein or post-translational modification. The mass shift does not relate to any single common chloroplast PTM (Lehtimäki et al., 2015); however, PTMs can have an important impact on Mb’s tertiary structure, for example cysteine S-nitrosation increases the heme’s affinity for oxygen (Helbo et al., 2014). CD confirms correct folding for Mb as an alpha helical protein (Almeida Ribeiro et al., 2003). While the results indicate that the recombinant Mb adopts a structure consistent with correctly folded Mb, additional studies would help determine the extent to which its functional properties match those of native Mb. Future work should include biochemical characterisation of ligand binding, assessment of redox behaviour, and evaluation of colour and flavour performance in food-relevant systems (Carlsson et al., 2020).

As a yield of fresh weight (FW), the Mb yield translated to an estimate of 94 ± 12 mg/kg for tobacco (assuming an average of 2.7% TSP) and 48 ± 2.6 mg/kg for lettuce (assuming 1.5% TSP). As for yield of dry weight (DW), this corresponded to 800 ± 110 mg/kg for tobacco and 810 ± 60 mg/kg for lettuce. Since quantification was performed on a limited number of biological samples in the present study, the reported quantification values should be considered approximate estimates of protein accumulation. Furthermore, antibody-based quantification may be influenced by factors like epitope accessibility; independent quantification approaches would therefore be valuable for validating recombinant Mb accumulation levels in future studies. Despite the lower accumulation in lettuce as a function of total soluble protein, its higher water content means that the dry weight Mb yields between the two plants are similar. This is a higher accumulation than previously reported for the *C. reinhardtii* chloroplast (44 mg/kg FW, 234 mg/kg of dry cell weight) (Phadnis and Prakash, 2024) but still lower than the purification yield from transient expression in *N. benthamiana* (~210 mg/kg FW from agroinfiltration, 60–80 mg/kg FW from agrospray) (Carlsson et al., 2020).

*C. reinhardtii* exhibited very low levels of bovine Mb protein accumulation (<0.25% TSP;<0.1 mg/L), and the doublet band in the immunoblot indicated an unexpected lower band, likely due to Mb degradation. These values were lower than those reported in previous literature (0.257 mg/L) (Phadnis and Prakash, 2024), likely due to the difference in strain or regulatory regions used. The data in this previous report did not show a doublet band in the immunoblot, likely due to the use of an anti-FLAG antibody as opposed to the anti-Mb antibody used here. It should be noted that, in the work presented here, different Mb orthologs were evaluated in the plant and algal expression systems: intrinsic differences between the orthologs may also influence protein accumulation, stability, or heme incorporation. Therefore, the present study demonstrates the feasibility of recombinant Mb production in each host but does not permit a direct quantitative comparison of expression performance across platforms. Future work using a single Mb ortholog across all hosts would allow a more rigorous assessment of host-dependent expression characteristics.

As a comparison with edible animal meat, bovine Mb yield amounts to 8.10 mg/g (8100 mg/kg) dry tissue from psoas major muscle (used for tenderloin/filet mignon) and 11.16 mg/g (11160 mg/kg) dry tissue from longissimus dorsi muscle (used for ribeye steak) (Rickansrud and Henrickson, 1967). Thus, the difference in dry weight Mb accumulation is around 10-fold higher in animal muscle compared with the plant yields described in this report. Despite this, plant cultivation is far more resource efficient than livestock production (Poore and Nemecek, 2018); consequently, plant-derived Mb could achieve protein yields per hectare that rival—or even potentially exceed—those of animal agriculture, whilst also benefiting from substantially lower water use and greenhouse gas emissions (Poore and Nemecek, 2018; Merlo et al., 2024). A dedicated techno-economic analysis (TEA), which is beyond the scope of the present study, could define the exact range of Mb expression level that would be competitive for large-scale, sustainable production. It should also be noted that detailed analyses were conducted using a single independent transplastomic line. Although plastid transformation employs site-specific homologous recombination and usually results in consistent transgene expression amongst transformant lines (Daniell et al., 2002), phenotypic variation arising from tissue culture, regeneration, or somaclonal variation cannot be entirely excluded (Basso et al., 2020). Validation of the observed phenotype in additional independently derived transplastomic lines would further strengthen the generality of these conclusions.

Nuclear accumulation of Mb protein in tobacco using the strong constitutive promoters from the cauliflower mosaic virus 35S or the *A. thaliana* ubiquitin 10 promoter was consistently lower than chloroplast expression in tobacco. Previous work in the literature comparing nuclear and tobacco expression of human somatotropin (hST) protein in tobacco has observed a similar result (Staub et al., 2000). Total heme levels were elevated two-fold in Nt-pAG108, which can be partially accounted for by the expression of Mb; from 35% heme-bound Mb with a yield of 94 mg/kg FW, at least an extra 1.93 μmol/kg FW of heme would be expected (5.5 μmol/kg FW for 100% heme-bound Mb), whereas an increase of 6 μmol/kg FW of heme is observed. Nevertheless, only 35% of the Mb was isolated with bound heme. Similarly, 20% heme binding was observed for porcine Mb expressed in *S. cerevisiae* (Yu et al., 2023).

In the present study, incomplete holo-Mb formation could be due to the expression of an exogenous hemoprotein such as Mb exceeding the capacity of the endogenous heme biosynthetic pathway (Culbertson and Olson, 2010). Competition with chlorophyll biosynthesis for shared tetrapyrrole precursors and inefficient heme insertion into apo-Mb may further contribute to the low heme occupancy. The limitation in heme supply may not only reduce holo-Mb formation but could also constrain overall Mb accumulation, as heme availability is important for the stability and proper folding of the protein (Culbertson and Olson, 2010). Heme accumulation strategies to improve Mb heme loading include engineering plant lines for heme accumulation, for example by overexpressing the terminal enzyme of heme *b* biosynthesis ferrochelatase 1 (FC1) (Page et al., 2020). Alternatively, supplementation of heme precursors, like aminolevulinic acid (ALA) (Wen and Grimm, 2024), could also improve the Mb heme loading by increasing the flux of the chloroplast tetrapyrrole pathway (Tanaka and Tanaka, 2007). The relatively low heme occupancy would most likely compromise downstream applications of plant-produced Mb, due to heme’s important contributions to the colour, taste, and nutritional properties of Mb (Suman and Joseph, 2013). Therefore, improving heme availability and incorporation is likely to be an important step towards realizing the full functional potential of plant-produced Mb. As mentioned, heme accumulation could increase Mb yield; other strategies for Mb accumulation could include the use of different regulatory regions and insertion sites, or inducible expression systems (Xu et al., 2024).

There exists a complicated regulatory network for tetrapyrrole synthesis in plants, where heme is considered the primary plastid signal that modulates nuclear gene expression, particularly for the induction of photosynthesis-associated nuclear genes (PhANGs) (Nagahatenna et al., 2015). Heme is also involved in many other processes in plants, such as the mitochondrial respiratory and chloroplast photosynthetic electron transport chains (Fang et al., 2024), and has roles in other contexts such as buffering oxygen in symbiotic nitrogen-fixing plants (Ott et al., 2005). Despite the complex relationship between heme and other plant processes, photosynthetic parameters (Y(II), ETR, and Y(NPQ)) were similar across the three genotypes, suggesting that heme binding to Mb did not impact photosynthetic processes. This is the first evidence that hemoprotein expression in tobacco chloroplasts had no drastic impact on growth or on photosynthetic performance, in part because heme levels were upregulated. While a previous publication described growth defects and reduced photosynthetic fitness in transplastomic tobacco expressing membrane-bound cytochrome P450 enzymes (Gnanasekaran et al., 2016), it was unclear from this previous work whether these effects were due to changes in heme levels, or the nature of the protein expressed. Other work has also shown that reduced heme levels in the homozygous null ferrochelatase 2 (FC2) T-DNA insertion mutant *fc2-2* (Espinas et al., 2016) led to a reduction in the overall chlorophyll content and chlorophyll *a* to *b* ratio, and impaired NPQ and Y(II).

Overexpression of hemoproteins in plants can have a variety of effects, depending on the protein: for example, heterologous expression of chloroplast-targeted *Vitreoscilla* hemoglobin in *Hyoscyamus niger* showed no significant difference in growth between control and transgenic plants (Guo et al., 2018), whereas the ectopic expression of chloroplast-targeted soybean leghemoglobin in potato displayed a dwarf phenotype, possibly due to gibberellin limitation (Bonna et al., 2008). Although a growth delay was observed in this work for the tobacco plant expressing Mb (Nt-pAG108) compared with Nt-wt, a similar effect was also identified in the control plant (Nt-pKP9), which would suggest that the transgene insertion site may not be completely neutral.

As for chlorophyll, Nt-pKP9 and Nt-pAG108 plants showed a significant increase compared with Nt-wt, which could also be related to the choice of insertion site. The chlorophyll *a* to chlorophyll *b* ratio appears equivalent between Nt-wt and Nt-pKP9, yet there was a slight decrease for Nt-pAG108. In Arabidopsis, knockdown of the heme-binding protein AtHBP5 resulted in a 25% increase in heme content, yet the chlorophyll content and chlorophyll *a* to *b* ratio remain unchanged (Lee et al., 2012). One limitation of the work presented here is that the detailed physiological analyses, including photosynthetic performance and total heme quantification, were conducted only in tobacco, the primary system for characterisation. Although no obvious phenotypic differences were observed in lettuce expressing Mb, we cannot exclude effects on plant physiology and metabolism. Given the relevance of lettuce as a potential food-production host, future studies should assess plant physiology in transformant lettuce and determine the effects of recombinant Mb accumulation.

Aside from heme’s practical role in plants, heme is also an important functional molecule in the human diet, meaning Mb can be useful as an additive for plant-based food; in fact, there has been much commercial interest regarding animal protein expression in plants, although very few reports have been published in the literature (Tusé et al., 2024). With increasing interest and concern about the effects of animal agriculture, the work presented here is particularly timely; successful expression of Mb in plants represents a significant step forwards in the development of plant-based ingredients that can replicate the sensory and nutritional qualities of animal-derived products. For recombinant protein production, plants offer several advantages over other organisms; they are highly scalable, are sustainable, and carry no human pathogens (Desai et al., 2010). Compared with plants, animal agriculture is an intrinsically inefficient process: the energy conversion of plant foods to foods of animal origin is estimated to be around 10:1 (Sabaté and Soret, 2014). Using non-allergenic edible crop hosts such as lettuce allows for less stringent purification protocols and regulation hurdles (Desai et al., 2010), as the biomass can be incorporated directly into food matrices with minimal or no purification; this offers potential advantages for scalability and cost. Regulatory frameworks with ethical, religious, and environmental considerations for the novel area of plant-produced animal proteins are still evolving and are yet to be defined by regulatory bodies (Bobo, 2024; Tusé et al., 2024).

Microbial systems, on the other hand, currently benefit from more established industrial fermentation infrastructure, more predictable product yields, and more streamlined regulatory pathways in certain jurisdictions (Gnaim et al., 2026). Plant-based protein production systems, including chloroplast-based platforms, are still nascent compared with well-established microbial expression systems, which benefit from decades of optimisation, industrial scale-up, and regulatory experience (Gnaim et al., 2026). While the threshold for the commercialisation of recombinant protein production in plants is usually around 0.1%-1% TSP (Twyman et al., 2003), the expression levels required for commercial deployment are highly dependent on the target application, production system, and downstream processing strategy. For Mb, commercial viability will depend not only on total protein accumulation but also on the yield of functional holo-Mb. Consequently, further improvements in both expression level and heme incorporation are likely to enhance the translational potential of the platform.

In summary, this work aligns with current consumer trends and regulatory shifts towards sustainability by advancing protein production in scalable, plant-based platforms. The promise of chloroplast transformation delivers overall in terms of higher protein expression than nuclear expression for Mb, but expression could be further enhanced by exploring heme optimisation strategies, different regulatory regions and growth conditions, and inducible expression systems.

## 5 Conclusions

In conclusion, the meat protein Mb has been successfully expressed in the chloroplast of *C. reinhardtii* (<0.25% TSP), tobacco (2.7% TSP), and lettuce (1.5% TSP). This is the first known report of heterologous hemoprotein expression in lettuce. The latter two are the first reports in the literature of stable Mb protein accumulation in higher plants. In *C. reinhardtii*, a doublet band is observed in immunoblots, probably due to protein degradation or cleavage. There is little evidence of oxidative damage from Mb expression in the tobacco chloroplast. Fresh weight yields (94 ± 12 mg/kg for tobacco and 48 ± 2.6 mg/kg for lettuce) and dry weight yields (800 ± 110 mg/kg for tobacco, 810 ± 60 mg/kg for lettuce) are promising but still require further optimisation to match animal muscle.

## Data Availability

The original contributions presented in the study are publicly available. This data can be found here: ENA (EBI). Accession for the study is: PRJEB120971 https://www.ebi.ac.uk/ena/browser/view/PRJEB120971.

## References

[B1] Adem M. Beyene D. Feyissa T. (2017). Recent achievements obtained by chloroplast transformation. Plant Methods 13, 30. doi: 10.1186/s13007-017-0179-1 28428810 PMC5395794

[B2] Almeida Ribeiro E. Regis W. C. B. Tasic L. Ramos C. H. I. (2003). Fast purification of the Apo form and of a non-binding heme mutant of recombinant sperm whale myoglobin. Protein Expr Purif. 28, 202–208. doi: 10.1016/s1046-5928(02)00651-4 12651126

[B3] Anomaly J. (2015). What's wrong with factory farming? Public Health Ethics 8, 246–254. doi: 10.1093/phe/phu001 36540869 PMC9757169

[B4] Apel W. Schulze W. X. Bock R. (2010). Identification of protein stability determinants in chloroplasts. Plant J. 63, 636–650. doi: 10.1111/j.1365-313x.2010.04268.x 20545891 PMC2988409

[B5] Barr I. Guo F. (2015). Pyridine hemochromagen assay for determining the concentration of heme in purified protein solutions. Bio Protoc. 5, e1594. doi: 10.21769/bioprotoc.1594 27390766 PMC4932910

[B6] Basso M. F. Arraes F. B. M. Grossi-de-Sa M. Moreira V. J. V. Alves-Ferreira M. Grossi-de-Sa M. F. (2020). Insights into genetic and molecular elements for transgenic crop development. Front. Plant Sci. 11, 509. doi: 10.3389/fpls.2020.00509 32499796 PMC7243915

[B7] Bobo J. (2024). Molecular farming navigates a complex regulatory landscape. Front. Plant Sci. 15, 1411943. doi: 10.3389/fpls.2024.1411943 39081529 PMC11286381

[B8] Bonna A. L. Chaparro-Giraldo A. Appezzato-da-Gloria B. Hedden P. Silva-Filho M. C. (2008). Ectopic expression of soybean leghemoglobin in chloroplasts impairs gibberellin biosynthesis and induces dwarfism in transgenic potato plants. Mol. Breed. 22, 613–618. doi: 10.1007/s11032-008-9203-5 30311153

[B9] Bowman S. E. Bren K. L. (2008). The chemistry and biochemistry of heme c: functional bases for covalent attachment. Nat. Prod. Rep. 25, 1118–1130. doi: 10.1039/b717196j 19030605 PMC2654777

[B10] Carlsson M. L. R. Kanagarajan S. Bülow L. Zhu L.-H. (2020). Plant based production of myoglobin - a novel source of the muscle heme-protein. Sci. Rep. 10, 920. doi: 10.1038/s41598-020-57565-y 31969582 PMC6976567

[B11] Castiglia D. Sannino L. Marcolongo L. Ionata E. Tamburino R. De Stradis A. . (2016). High-level expression of thermostable cellulolytic enzymes in tobacco transplastomic plants and their use in hydrolysis of an industrially pretreated Arundo donax L. biomass. Biotechnol. Biofuels 9, 154. doi: 10.1186/s13068-016-0569-z 27453729 PMC4957871

[B12] Chen Y. Nishimura K. Tokizawa M. Yamamoto Y. Y. Oka Y. Matsushita T. . (2024). Alternative localization of HEME OXYGENASE 1 in plant cells regulates cytosolic heme catabolism. Plant Physiol. 195, 2937–2951. doi: 10.1093/plphys/kiae288 38805221

[B13] Culbertson D. S. Olson J. S. (2010). Role of heme in the unfolding and assembly of myoglobin. Biochemistry. 49, 6052–6063. doi: 10.1021/bi1006942 20540498 PMC2912444

[B14] Cutruzzolà F. Travaglini Allocatelli C. Brancaccio A. Brunori M. (1996). Aplysia limacina myoglobin cDNA cloning: an alternative mechanism of oxygen stabilization as studied by active-site mutagenesis. Biochem. J. 314, 83–90. doi: 10.1042/bj3140083 8660313 PMC1217055

[B15] Daniell H. Khan M. S. Allison L. (2002). Milestones in chloroplast genetic engineering: an environmentally friendly era in biotechnology. Trends Plant Sci. 7, 84–91. doi: 10.1016/s1360-1385(01)02193-8 11832280 PMC3476055

[B16] Davoodi-Semiromi A. Schreiber M. Nalapalli S. Verma D. Singh N. D. Banks R. K. . (2010). Chloroplast-derived vaccine antigens confer dual immunity against cholera and malaria by oral or injectable delivery. Plant Biotechnol. J. 8, 223–242. doi: 10.1111/j.1467-7652.2009.00479.x 20051036 PMC2807910

[B17] Desai P. N. Shrivastava N. Padh H. (2010). Production of heterologous proteins in plants: strategies for optimal expression. Biotechnol. Adv. 28, 427–435. doi: 10.1016/j.bioteChadv.2010.01.005 20152894

[B18] Engler C. Kandzia R. Marillonnet S. (2008). A one pot, one step, precision cloning method with high throughput capability. PLoS One 3, e3647. doi: 10.1371/journal.pone.0003647 18985154 PMC2574415

[B19] Espinas N. A. Kobayashi K. Sato Y. Mochizuki N. Takahashi K. Tanaka R. . (2016). Allocation of heme is differentially regulated by ferrochelatase isoforms in Arabidopsis cells. Front. Plant Sci. 7, 1326. doi: 10.3389/fpls.2016.01326 27630653 PMC5005420

[B20] Espinas N. A. Kobayashi K. Takahashi S. Mochizuki N. Masuda T. (2012). Evaluation of unbound free heme in plant cells by differential acetone extraction. Plant Cell Physiol. 53, 1344–1354. doi: 10.1093/pcp/pcs067 22555813

[B21] Fairweather-Tait S. (2023). The role of meat in iron nutrition of vulnerable groups of the UK population. Front. Anim. Sci. 4, 1142252. doi: 10.3389/fanim.2023.1142252

[B22] Fang Y. Tai Z. Hu K. Luo L. Yang S. Liu M. . (2024). Comprehensive review on plant cytochrome P450 evolution: copy number, diversity, and motif analysis from Chlorophyta to Dicotyledoneae. Genome Biol. Evol. 16, evae240. doi: 10.1093/gbe/evae240 39506518 PMC11586672

[B23] Fatima I. Wakade G. Ahmad N. Daniell H. (2025). Expression of endochitinase and exochitinase in lettuce chloroplasts increases plant biomass and kills fungal pathogen Candida albicans. Plant Biotechnol. J. 23, 1437–1451. doi: 10.1111/pbi.14596 39967296 PMC12018847

[B24] Foyer C. H. Hanke G. (2022). ROS production and signalling in chloroplasts: cornerstones and evolving concepts. Plant J. 111, 642–661. doi: 10.1111/tpj.15856 35665548 PMC9545066

[B25] Gisby M. F. Mellors P. Madesis P. Ellin M. Laverty H. O'Kane S. . (2011). A synthetic gene increases TGFβ3 accumulation by 75-fold in tobacco chloroplasts enabling rapid purification and folding into a biologically active molecule. Plant Biotechnol. J. 9, 618–628. doi: 10.1111/j.1467-7652.2011.00619.x 21535357

[B26] Gnaim R. Kassim H. Neidhardt L. Gassler T. Ledesma-Amaro R. (2026). Navigating adoption barriers for microbial proteins in future food. Nat. Commun. 17, 5279. doi: 10.1038/s41467-026-73987-0 42270635 PMC13269705

[B27] Gnanasekaran T. Karcher D. Nielsen A. Z. Martens H. J. Ruf S. Kroop X. . (2016). Transfer of the cytochrome P450-dependent dhurrin pathway from Sorghum bicolor into Nicotiana tabacum chloroplasts for light-driven synthesis. J. Exp. Bot. 67, 2495–2506. doi: 10.1093/jxb/erw067 26969746 PMC4809297

[B28] Gorman D. S. Levine R. P. (1965). Cytochrome f and plastocyanin: their sequence in the photosynthetic electron transport chain of Chlamydomonas reinhardi. Proc. Natl. Acad. Sci. U.S.A. 54, 1665–1669. doi: 10.1073/pnas.54.6.1665 4379719 PMC300531

[B29] Gray B. N. Bougri O. Carlson A. R. Meissner J. Pan S. Parker M. H. . (2011). Global and grain-specific accumulation of glycoside hydrolase family 10 xylanases in transgenic maize (Zea mays). Plant Biotechnol. J. 9, 1100–1108. doi: 10.1111/j.1467-7652.2011.00632.x 21689368

[B30] Grefen C. Donald N. Hashimoto K. Kudla J. Schumacher K. Blatt M. R. (2010). A ubiquitin-10 promoter-based vector set for fluorescent protein tagging facilitates temporal stability and native protein distribution in transient and stable expression studies. Plant J. 64, 355–365. doi: 10.1111/j.1365-313X.2010.04322.x 20735773

[B31] Guo Z. Tan H. Lv Z. Ji Q. Huang Y. Liu J. . (2018). Targeted expression of Vitreoscilla hemoglobin improves the production of tropane alkaloids in Hyoscyamus Niger hairy roots. Sci. Rep. 8, 17969. doi: 10.1038/s41598-018-36156-y 30568179 PMC6299274

[B32] Helbo S. Gow A. J. Jamil A. Howes B. D. Smulevich G. Fago A. (2014). Oxygen-linked S-nitrosation in fish myoglobins: a cysteine-specific tertiary allosteric effect. PLoS One 9, e97012. doi: 10.1371/journal.pone.0097012 24879536 PMC4039430

[B33] Jareonsin S. Pumas C. (2021). Advantages of heterotrophic microalgae as a host for phytochemicals production. Front. Bioeng. Biotechnol. 9, 628597. doi: 10.3389/fbioe.2021.628597 33644020 PMC7907617

[B34] Kanamoto H. Yamashita A. Asao H. Okumura S. Takase H. Hattori M. . (2006). Efficient and stable transformation of Lactuca sativa L. cv. Cisco (lettuce) plastids. Transgenic Res. 15, 205–217. doi: 10.1007/s11248-005-3997-2 16604461

[B35] Kendrew J. C. Bodo G. Dintzis H. M. Parrish R. G. Wyckoff H. Phillips D. C. (1958). A three-dimensional model of the myoglobin molecule obtained by x-ray analysis. Nature 181, 662–666. doi: 10.1038/181662a0 13517261

[B36] Koneswaran G. Nierenberg D. (2008). Global farm animal production and global warming: impacting and mitigating climate change. Environ. Health Perspect. 116, 578–582. doi: 10.1289/ehp.11034 18470284 PMC2367646

[B37] Lee H. J. Mochizuki N. Masuda T. Buckhout T. J. (2012). Disrupting the bimolecular binding of the haem-binding protein 5 (AtHBP5) to haem oxygenase 1 (HY1) leads to oxidative stress in Arabidopsis. J. Exp. Bot. 63, 5967–5978. doi: 10.1093/jxb/ers242 22991161 PMC3467301

[B38] Lehtimäki N. Koskela M. M. Mulo P. (2015). Posttranslational modifications of chloroplast proteins: an emerging field. Plant Physiol. 168, 768–775. doi: 10.1104/pp.15.00117 25911530 PMC4741338

[B39] Lichtenthaler H. K. Wellburn A. R. (1983). Determinations of total carotenoids and chlorophylls a and b of leaf extracts in different solvents. Biochem. Soc Trans. 11, 591–592. doi: 10.1042/bst0110591

[B40] Lurie-Luke E. (2024). Alternative protein sources: science powered startups to fuel food innovation. Nat. Commun. 15, 4425. doi: 10.1038/s41467-024-47091-0 38806477 PMC11133469

[B41] Lutz K. A. Svab Z. Maliga P. (2006). Construction of marker-free transplastomic tobacco using the Cre-loxP site-specific recombination system. Nat. Protoc. 1, 900–910. doi: 10.1038/nprot.2006.118 17406323

[B42] Maliga P. Tungsuchat-Huang T. Lutz K. A. (2021). “ Transformation of the plastid genome in tobacco: the model system for chloroplast genome engineering,” in Chloroplast Biotechnology: Methods and Protocols. Ed. Maliga P. ( Springer US, New York, NY), 135–153. 10.1007/978-1-0716-1472-3_6 34028766

[B43] Meng Y. Xie L. You K. Chen W. (2023). High-level secretory production of leghemoglobin and myoglobin in Escherichia coli through inserting signal peptides. Food Biosci. 56, 103356. doi: 10.1016/j.fbio.2023.103356 38826717

[B44] Merlo M. Hennessy T. Buckley C. O'Mahony J. (2024). A comparison of animal and plant-based proteins from an economic, environmental, and nutritional perspective in the Republic of Ireland. Agric. Syst. 221, 104143. doi: 10.1016/j.agsy.2024.104143 38826717

[B45] Millar S. J. Moss B. W. Stevenson M. H. (1996). Some observations on the absorption spectra of various myoglobin derivatives found in meat. Meat Sci. 42, 277–288. doi: 10.1016/0309-1740(94)00045-x 22060775

[B46] Mitiouchkina T. Mishin A. S. Somermeyer L. G. Markina N. M. Chepurnyh T. V. Guglya E. B. . (2020). Plants with genetically encoded autoluminescence. Nat. Biotechnol. 38, 944–946. doi: 10.1038/s41587-020-0500-9 32341562 PMC7610436

[B47] Mok Y. G. Hong S. Seo D. I. Choi S. Kim H. K. Jin D. M. . (2024). Herbicide-resistant plants produced by precision adenine base editing in plastid DNA. Nat. Plants 10, 1652–1658. doi: 10.1038/s41477-024-01808-7 39327461

[B48] Nagahatenna D. S. Langridge P. Whitford R. (2015). Tetrapyrrole-based drought stress signalling. Plant Biotechnol. J. 13, 447–459. doi: 10.1111/pbi.12356 25756609 PMC5054908

[B49] Nakamura Y. Gojobori T. Ikemura T. (2000). Codon usage tabulated from international DNA sequence databases: status for the year 2000. Nucleic Acids Res. 28, 292. doi: 10.1093/nar/28.1.292 10592250 PMC102460

[B50] National Academies of Sciences, Engineering, and Medicine . (2023). “ Alternative protein sources: balancing food innovation, sustainability, nutrition, and health: proceedings of a workshop,” in The National Academies Collection: Reports Funded by National Institutes of Health. Ed. Nicholson A. ( National Academies Press (US, Washington, DC). 37229237

[B51] Neupert J. Karcher D. Bock R. (2008). Design of simple synthetic RNA thermometers for temperature-controlled gene expression in Escherichia coli. Nucleic Acids Res. 36, e124. doi: 10.1093/nar/gkn545 18753148 PMC2577334

[B52] Oey M. Lohse M. Kreikemeyer B. Bock R. (2009). Exhaustion of the chloroplast protein synthesis capacity by massive expression of a highly stable protein antibiotic. Plant J. 57, 436–445. doi: 10.1111/j.1365-313x.2008.03702.x 18939966

[B53] Ott T. van Dongen J. T. Günther C. Krusell L. Desbrosses G. Vigeolas H. . (2005). Symbiotic leghemoglobins are crucial for nitrogen fixation in legume root nodules but not for general plant growth and development. Curr. Biol. 15, 531–538. doi: 10.1016/j.cub.2005.01.042 15797021

[B54] Page M. T. Garcia-Becerra T. Smith A. G. Terry M. J. (2020). Overexpression of chloroplast-targeted ferrochelatase 1 results in a genomes uncoupled chloroplast-to-nucleus retrograde signalling phenotype. Philos. Trans. R. Soc Lond. B. Biol. Sci. 375, 20190401. doi: 10.1098/rstb.2019.0401 32362255 PMC7209946

[B55] Paul K. Theorell H. Åkeson Å. Virtanen A. Sörensen N. (1953). The molar light absorption of pyridine ferroprotoporphrin (pyridine haemochromogen). Acta Chem. Scand. 7, 1284–1287. doi: 10.3891/acta.chem.scand.07-1284

[B56] Phadnis G. Prakash G. (2024). Chloroplast expression of myoglobin and functional characterization of C. reinhardtii biomass for alternative meat applications. Algal Res. 84, 103807. doi: 10.1016/j.algal.2024.103807 38826717

[B57] Phillips G. N. Jr. Arduini R. M. Springer B. A. Sligar S. G. (1990). Crystal structure of myoglobin form a synthetic gene. Proteins. 7, 358–365. doi: 10.1002/prot.340070407 2199973

[B58] Poore J. Nemecek T. (2018). Reducing food’s environmental impacts through producers and consumers. Science. 360, 987–992. doi: 10.1126/science.aaq0216 29853680

[B59] Puigbò P. Guzmán E. Romeu A. Garcia-Vallvé S. (2007). OPTIMIZER: a web server for optimizing the codon usage of DNA sequences. Nucleic Acids Res. 35, W126–W131. doi: 10.1093/nar/gkm219 17439967 PMC1933141

[B60] Rasala B. A. Muto M. Sullivan J. Mayfield S. P. (2011). Improved heterologous protein expression in the chloroplast of Chlamydomonas reinhardtii through promoter and 5′ untranslated region optimization. Plant Biotechnol. J. 9, 674–683. doi: 10.1111/j.1467-7652.2011.00620.x 21535358

[B61] Rickansrud D. A. Henrickson R. L. (1967). Total pigments and myoglobin concentration in four bovine muscles. J. Food Sci. 32, 57–61. doi: 10.1111/j.1365-2621.1967.tb01957.x 40046247

[B62] Rousseaux J. Dautrevaux M. Han K. (1976). Comparison of the amino acid sequence of pig heart myoglobin with other ungulate myoglobins. Biochim. Biophys. Acta 439, 55–62. doi: 10.1016/0005-2795(76)90160-4 952959

[B63] Ruhlman T. A. (2014). “ Plastid transformation in lettuce (Lactuca sativa L.) by biolistic DNA delivery,” in Chloroplast Biotechnology: Methods and Protocols. Ed. Maliga P. ( Humana Press, Totowa, NJ), 331–343. 10.1007/978-1-62703-995-6_21 24599864

[B64] Ruhlman T. Ahangari R. Devine A. Samsam M. Daniell H. (2007). Expression of cholera toxin B-proinsulin fusion protein in lettuce and tobacco chloroplasts--oral administration protects against development of insulitis in non-obese diabetic mice. Plant Biotechnol. J. 5, 495–510. doi: 10.1111/j.1467-7652.2007.00259.x 17490448 PMC2590789

[B65] Ruhlman T. Verma D. Samson N. Daniell H. (2010). The role of heterologous chloroplast sequence elements in transgene integration and expression. Plant Physiol. 152, 2088–2104. doi: 10.1104/pp.109.152017 20130101 PMC2850035

[B66] Ruiz O. N. Alvarez D. Gonzalez-Ruiz G. Torres C. (2011). Characterization of mercury bioremediation by transgenic bacteria expressing metallothionein and polyphosphate kinase. BMC Bio/Technol. 11, 82. doi: 10.1186/1472-6750-11-82 21838857 PMC3180271

[B67] Sabaté J. Soret S. (2014). Sustainability of plant-based diets: back to the future. Am. J. Clin. Nutr. 100, 476S–482S. doi: 10.3945/ajcn.113.071522 24898222

[B68] Santo R. E. Kim B. F. Goldman S. E. Dutkiewicz J. Biehl E. M. B. Bloem M. W. . (2020). Considering plant-based meat substitutes and cell-based meats: a public health and food systems perspective. Front. Sustain. Food. Syst. 4, 134. doi: 10.3389/fsufs.2020.00134

[B69] Schindelin J. Arganda-Carreras I. Frise E. Kaynig V. Longair M. Pietzsch T. . (2012). Fiji: an open-source platform for biological-image analysis. Nat. Methods 9, 676–682. doi: 10.1038/nmeth.2019 22743772 PMC3855844

[B70] Schnurr J. A. Guerra D. J. (2000). The CaMV-35S promoter is sensitive to shortened photoperiod in transgenic tobacco. Plant Cell Rep. 19, 279–282. doi: 10.1007/s002990050012 30754908

[B71] Sezonov G. Joseleau-Petit D. D'Ari R. (2007). Escherichia coli Physiology in Luria-Bertani Broth. J. Bacteriol. 189, 8746–49. doi: 10.1128/JB.01368-07 17905994 PMC2168924

[B72] Shimada H. Fukasawa T. Ishimura Y. (1989). Expression of bovine myoglobin cDNA as a functionally active holoprotein in Saccharomyces cerevisiae. J. Biochem. 105, 417–422. doi: 10.1093/oxfordjournals.jbchem.a122679 2659586

[B73] Skoog F. Miller C. O. (1957). Chemical regulation of growth and organ formation in plant tissues cultured *in vitro*. Symp. Soc Exp. Biol. 11, 118–130. 13486467

[B74] Smerdon S. J. Oldfield T. J. Dodson E. J. Dodson G. G. Hubbard R. E. Wilkinson A. J. (1990). Determination of the crystal structure of recombinant pig myoglobin by molecular replacement and its refinement. Acta Crystallogr. B. 46, 370–377. doi: 10.2210/pdb1pmb/pdb 2383370

[B75] Southern blotting: Capillary transfer of DNA to membranes (2004). Southern blotting: capillary transfer of DNA to membranes. Nat. Methods 1, 91–92. doi: 10.1038/nmeth1004-91

[B76] Staub J. M. Garcia B. Graves J. Hajdukiewicz P. T. J. Hunter P. Nehra N. . (2000). High-yield production of a human therapeutic protein in tobacco chloroplasts. Nat. Biotechnol. 18, 333–338. doi: 10.1038/73796 10700152

[B77] Suman S. P. Joseph P. (2013). Myoglobin chemistry and meat color. Annu. Rev. Food Sci. Technol. 4, 79–99. doi: 10.1146/annurev-food-030212-182623 23190143

[B78] Tanaka R. Tanaka A. (2007). Tetrapyrrole biosynthesis in higher plants. Annu. Rev. Plant Biol. 58, 321–346. doi: 10.1146/annurev.arplant.57.032905.105448 17227226

[B79] Taunt H. N. Jackson H. O. Gunnarsson Í.N. Pervaiz R. Purton S. (2023). Accelerating chloroplast engineering: a new system for rapid generation of marker-free transplastomic lines of Chlamydomonas reinhardtii. Microorganisms 11, 1967. doi: 10.3390/microorganisms11081967 37630526 PMC10457852

[B80] Taylor G. M. Mordaka P. M. Heap J. T. (2019). Start-stop assembly: a functionally scarless DNA assembly system optimized for metabolic engineering. Nucleic Acids Res. 47, e17. doi: 10.1093/nar/gky1182 30462270 PMC6379671

[B81] Tusé D. McNulty M. McDonald K. A. Buchman L. W. (2024). A review and outlook on expression of animal proteins in plants. Front. Plant Sci. 15, 1426239. doi: 10.3389/fpls.2024.1426239 39239203 PMC11374769

[B82] Twyman R. M. Stoger E. Schillberg S. Christou P. Fischer R. (2003). Molecular farming in plants: host systems and expression technology. Trends Biotechnol. 21, 570–578. doi: 10.1016/j.tibtech.2003.10.002 14624867

[B83] van Eerde A. Gottschamel J. Bock R. Hansen K. E. A. Munang'andu H. M. Daniell H. . (2019). Production of tetravalent dengue virus envelope protein domain III based antigens in lettuce chloroplasts and immunologic analysis for future oral vaccine development. Plant Biotechnol. J. 17, 1408–1417. doi: 10.1111/pbi.13065 30578710 PMC6576073

[B84] Varadarajan R. Szabo A. Boxer S. G. (1985). Cloning, expression in Escherichia coli, and reconstitution of human myoglobin. Proc. Natl. Acad. Sci. U.S.A. 82, 5681–5684. doi: 10.1073/pnas.82.17.5681 3898068 PMC390615

[B85] Verhounig A. Karcher D. Bock R. (2010). Inducible gene expression from the plastid genome by a synthetic riboswitch. Proc. Natl. Acad. Sci. U.S.A. 107, 6204–6209. doi: 10.1073/pnas.0914423107 20308585 PMC2852001

[B86] Wang Y. Fan J. Wei Z. Xing S. (2023). Efficient expression of fusion human epidermal growth factor in tobacco chloroplasts. BMC Bio/Technol. 23, 1. doi: 10.1186/s12896-022-00771-5 36611158 PMC9824920

[B87] Wen B. Grimm B. (2024). A genetically encoded fluorescent heme sensor detects free heme in plants. Plant Physiol. 196, 830–841. doi: 10.1093/plphys/kiae291 38762898 PMC11444292

[B88] Xu W. Li S. Bock R. Zhang J. (2024). A heat-inducible expression system for external control of gene expression in plastids. Plant Biotechnol. J. 22, 960–969. doi: 10.1111/pbi.14238 38059318 PMC10955493

[B89] Xue J. Zhou J. Li J. Du G. Chen J. Wang M. . (2023). Systematic engineering of Saccharomyces cerevisiae for efficient synthesis of hemoglobins and myoglobins. Bioresour. Technol. 370, 128556. doi: 10.1016/j.biortech.2022.128556 36586429

[B90] Yu F. Zhao X. Zhou J. Lu W. Li J. Chen J. . (2023). Biosynthesis of high-active hemoproteins by the efficient heme-supply Pichia pastoris chassis. Adv. Sci. 10, 2302826. doi: 10.1002/advs.202302826 37649147 PMC10602571

[B91] Zhou F. Badillo-Corona J. A. Karcher D. Gonzalez-Rabade N. Piepenburg K. Borchers A. M. . (2008). High-level expression of human immunodeficiency virus antigens from the tobacco and tomato plastid genomes. Plant Biotechnol. J. 6, 897–913. doi: 10.1111/j.1467-7652.2008.00356.x 19548344

