# Supplementary material for: Sustainable production of myoglobin meat protein in plant chloroplasts
Source: Front Plant Sci. 2026 Aug 6;17:1876707. doi: 10.3389/fpls.2026.1876707 (PMC13520740; doi:10.3389/fpls.2026.1876707)
Supplement: Supplementary file 1 [file DataSheet1.pdf]

Supplementary material for: **Sustainable production of myoglobin meat protein in plant chloroplasts**

This file includes:

Figures S1-9
Table S1-3

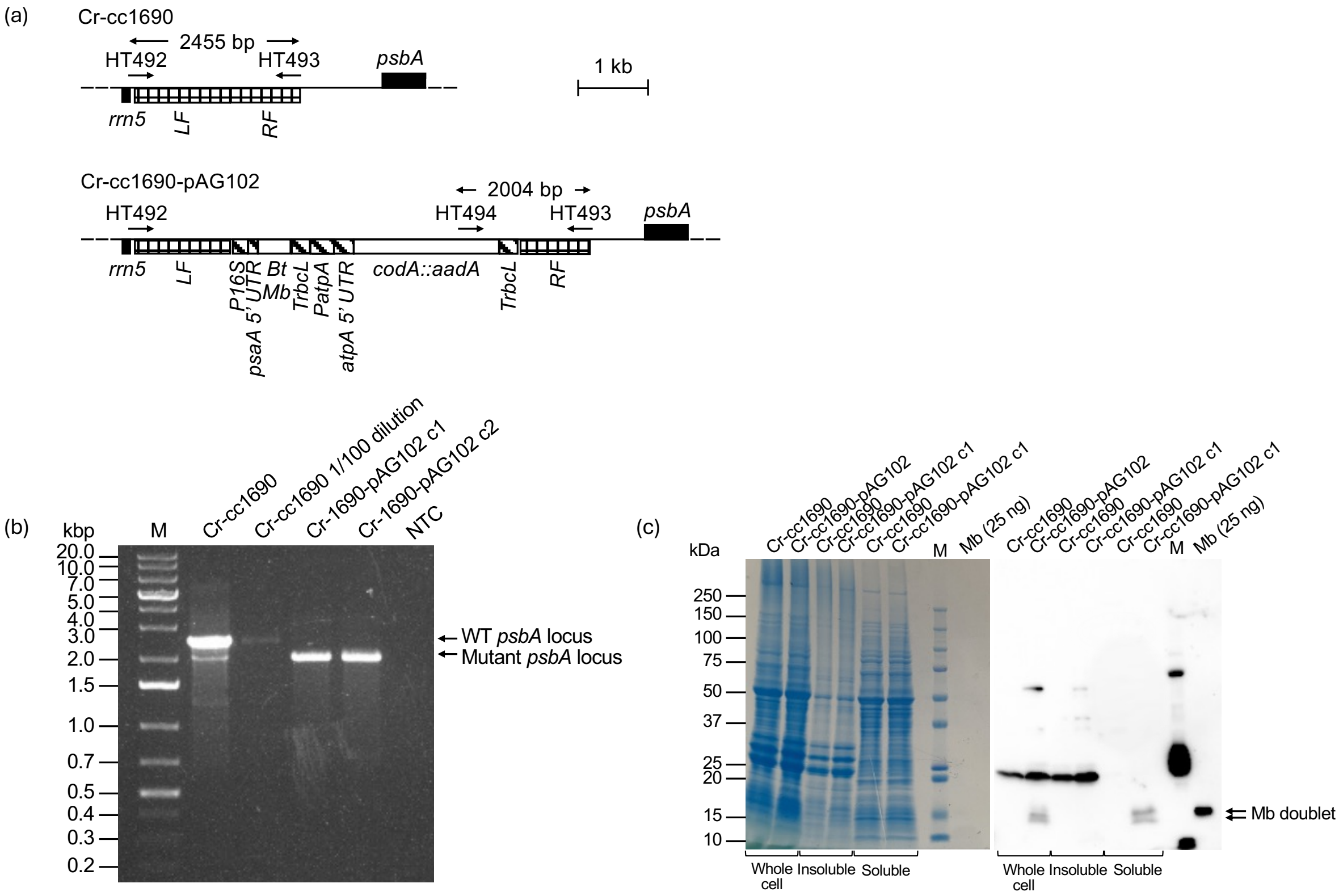

Figure S1. Generation of *Chlamydomonas reinhardtii* plastid transformants using vector pAG102 and analysis of mutants. (a) Map of the target region within the *C. reinhardtii* plastid genome and the design of vector pAG102 used for *C. reinhardtii* plastid transformation. The transgenes are targeted to the intergenic region between *rrn5* and *psbA*. The selectable marker gene *aadA* is driven by the *atpA* promoter (PatpA) and followed by the 3′ UTR from the plastid RuBisCO large subunit gene (TrbcL). The bovine myoglobin gene (*Bt Mb*) is driven by the 16S rRNA promoter (P16S) followed by the 5' UTR from the *psaA* gene and at the 3' end by the TrbcL terminator. (b) PCR analysis confirming homoplasmy of *C. reinhardtii* plastid transformants. Predicted PCR products are shown in (a). (c) Immunoblot of whole cell, insoluble and soluble fractions of *C. reinhardtii* strains. Immunoblotting using an α-Mb antibody (right) allows for Mb detection and quantification of expression as a proportion of total soluble protein (TSP) by comparing a known amount of Mb standard to the 10 μg of soluble protein loaded. The replicate stained SDS-PAGE gel (left) shows even loading.

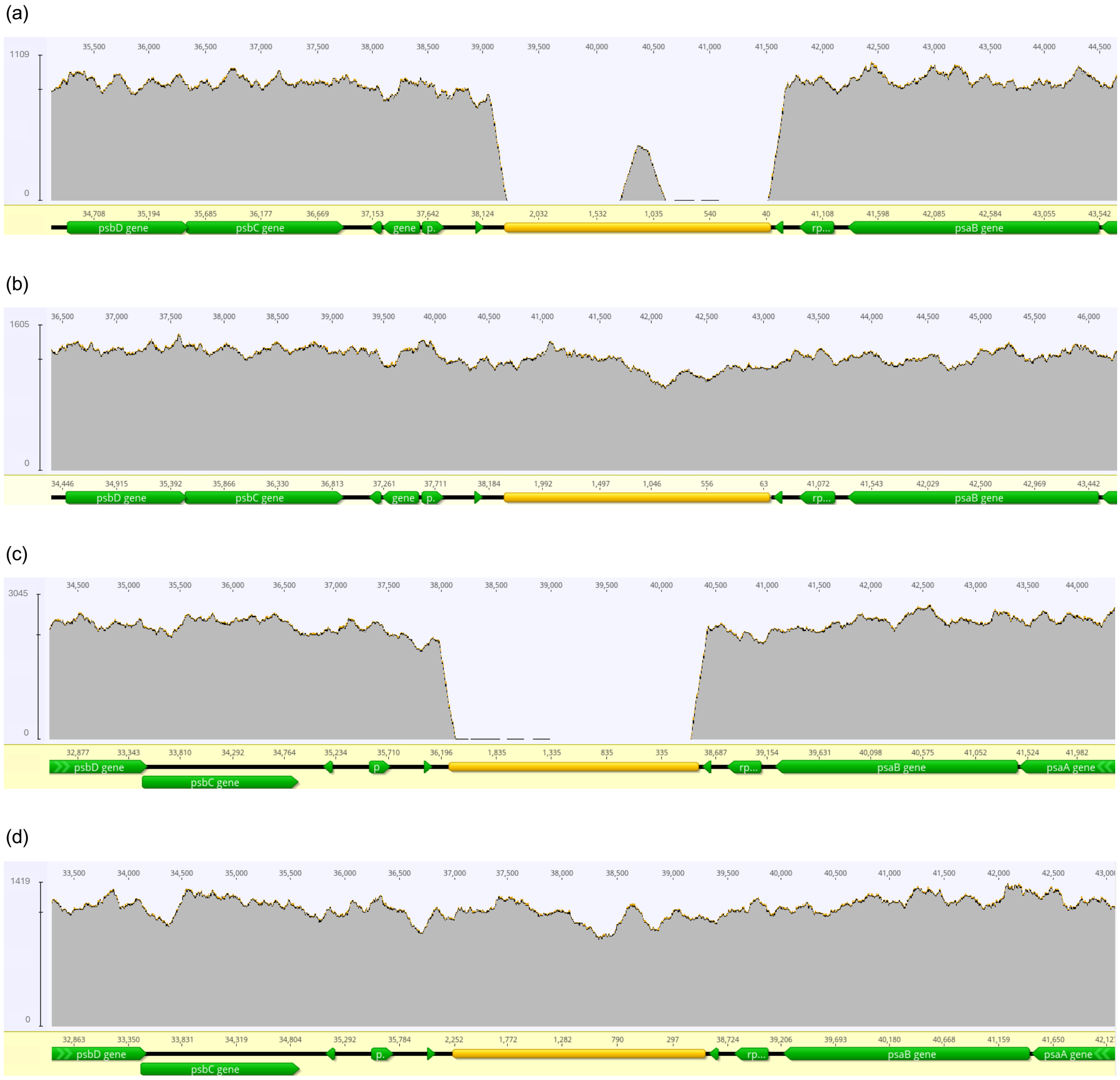

Figure S2. Sequencing data for tobacco and lettuce wild-type and transplastomic lines. (a) Wild-type tobacco (Nt-wt) sequencing reads aligned to the transplastomic tobacco genome (Nt-pAG108). The peak arising in the middle of the insertion originates from reads aligning to the *psbA* terminator. (b) Transplastomic tobacco (Nt-pAG108) sequencing reads aligned to the transplastomic tobacco genome (Nt-pAG108). (c) Wild-type lettuce (Ls-wt) sequencing reads aligned to the transplastomic lettuce genome (Ls-pAG115). (d) Transplastomic lettuce (Ls-pAG115) sequencing reads aligned to the transplastomic lettuce genome (Ls-pAG115). The vertical axis represents sequence coverage across the *psbD* to *psaA* locus of the chloroplast genome. The expected insertion sequence is shown in yellow.

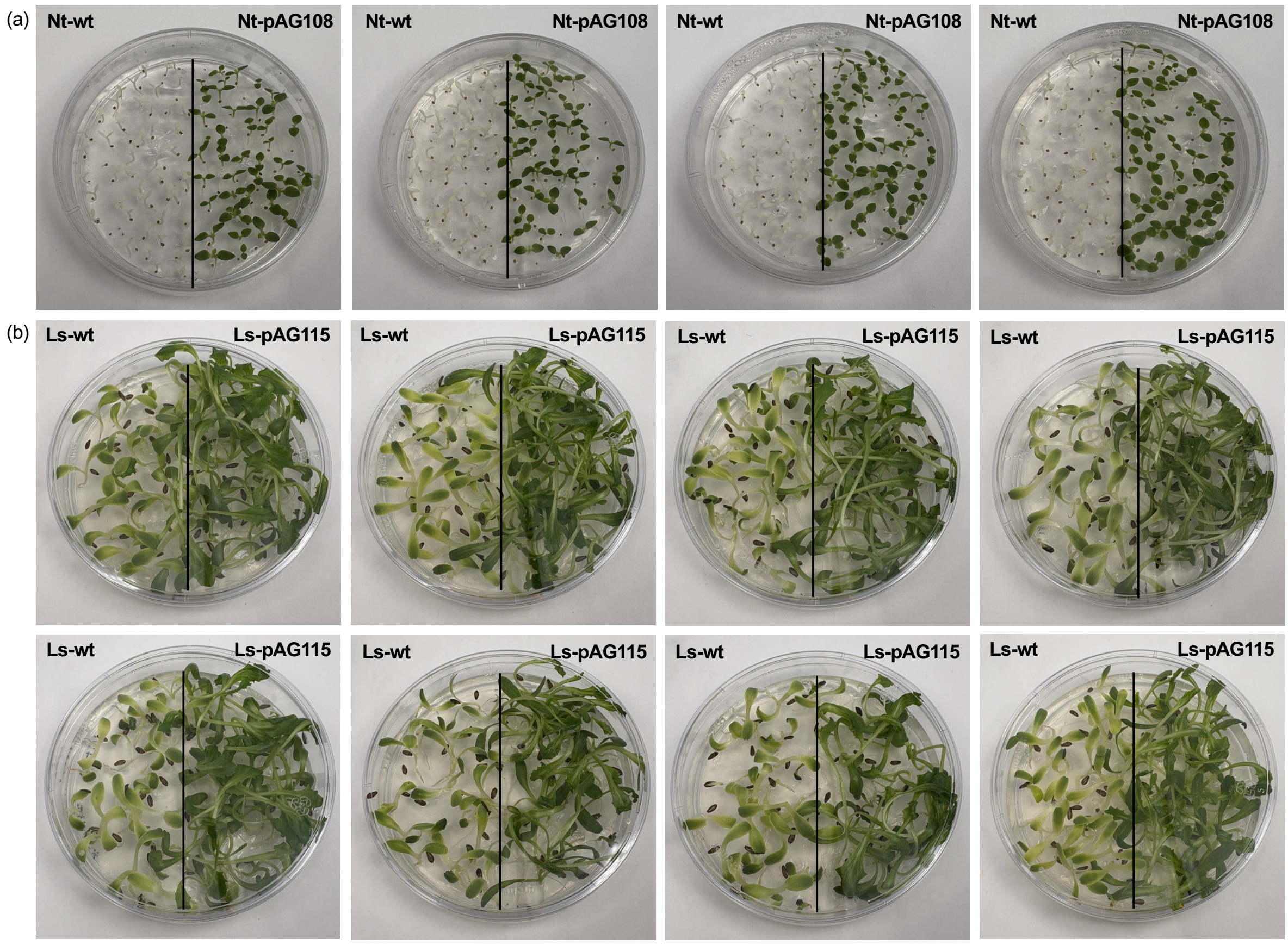

Figure S3. Large-scale inheritance and germination assay for tobacco and lettuce. (a) Nt-wt and Nt-pAG108 seeds sown on MS containing spectinomycin and streptomycin. 50 Nt-wt and 50 Nt-pAG108 seeds are sown per plate to measure bleaching and germination frequencies. (b) Ls-wt and Ls-pAG115 seeds sown on MS containing spectinomycin and streptomycin. 25 Ls-wt and 25 Ls-pAG115 seeds are sown per plate to measure bleaching and germination frequencies.

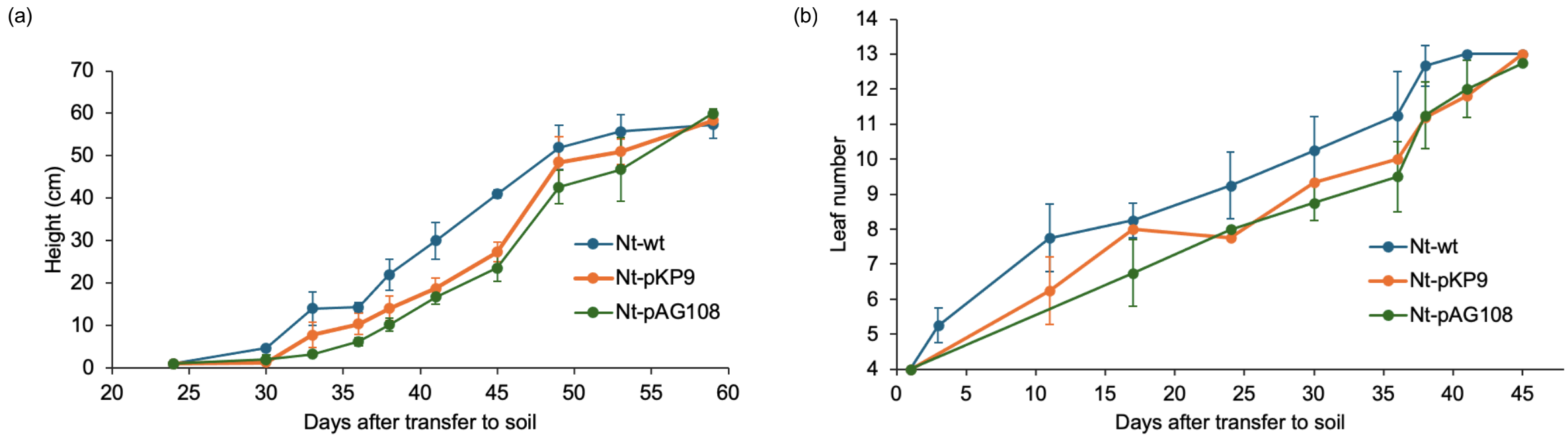

Figure S4. Growth assays for wild-type and transplastomic tobacco. (a) Height of plant as a function of days after transfer to soil. Height was measured for Nt-wt (blue), Nt-pKP9 (orange) and Nt-pAG108 (green). (b) Leaf number of plant as a function of days after transfer to soil. Leaf number was measured for Nt-wt (blue), Nt-pKP9 (orange) and Nt-pAG108 (green). These graphs were calculated from 5 biological replicates and error bars represent standard deviations.

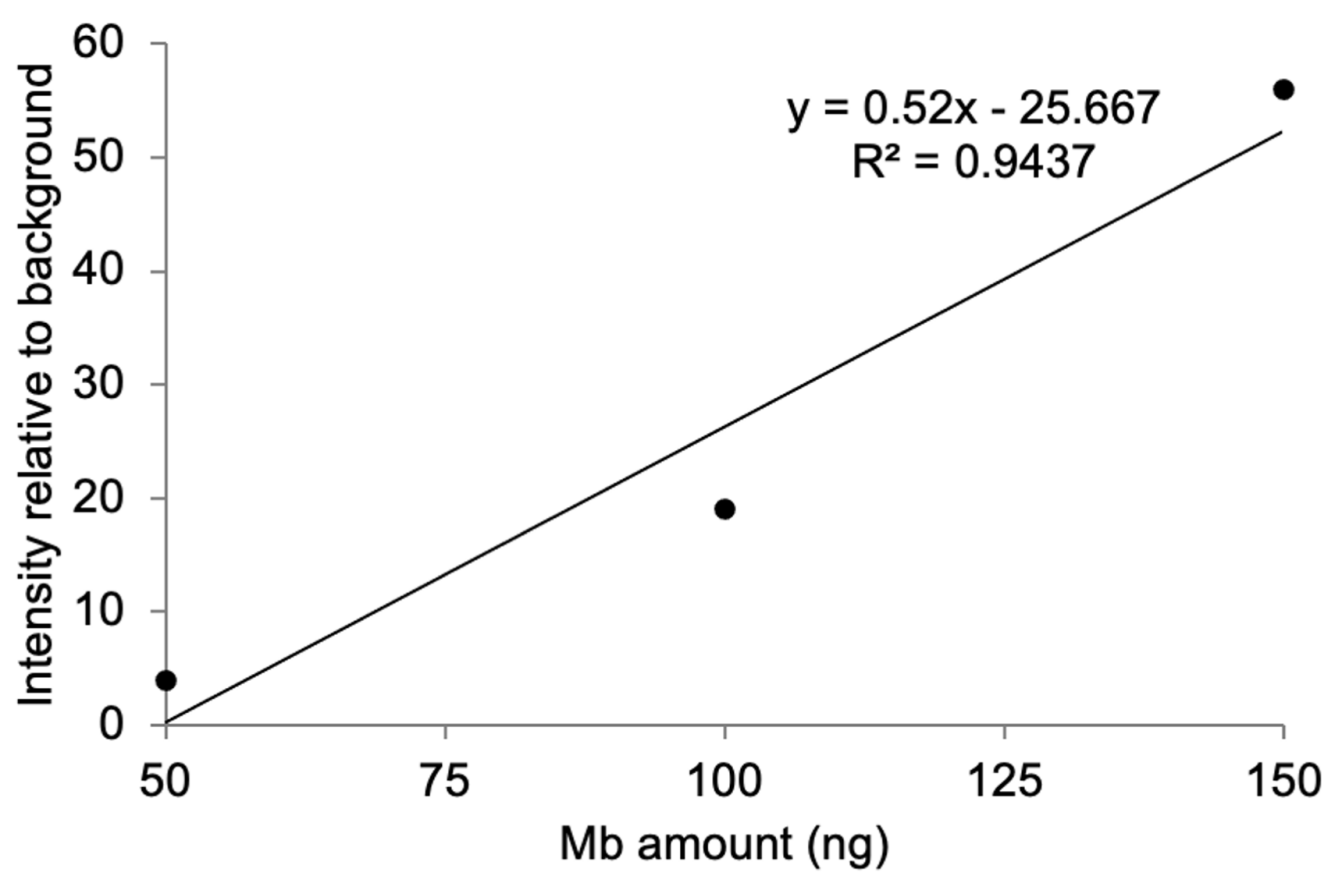

Figure S5. Standard curve for semi-quantification of porcine Mb expression from immunoblot in Figure 3. The immunoblot signals of the standard Mb bands from Figure 3b were measured relative to background for semi-quantitative quantification by densitometry of porcine Mb expression. The tobacco band signal corresponded to 140 ng Mb (2.8% TSP), and the lettuce band signal corresponded to 75 ng Mb (1.5% TSP).

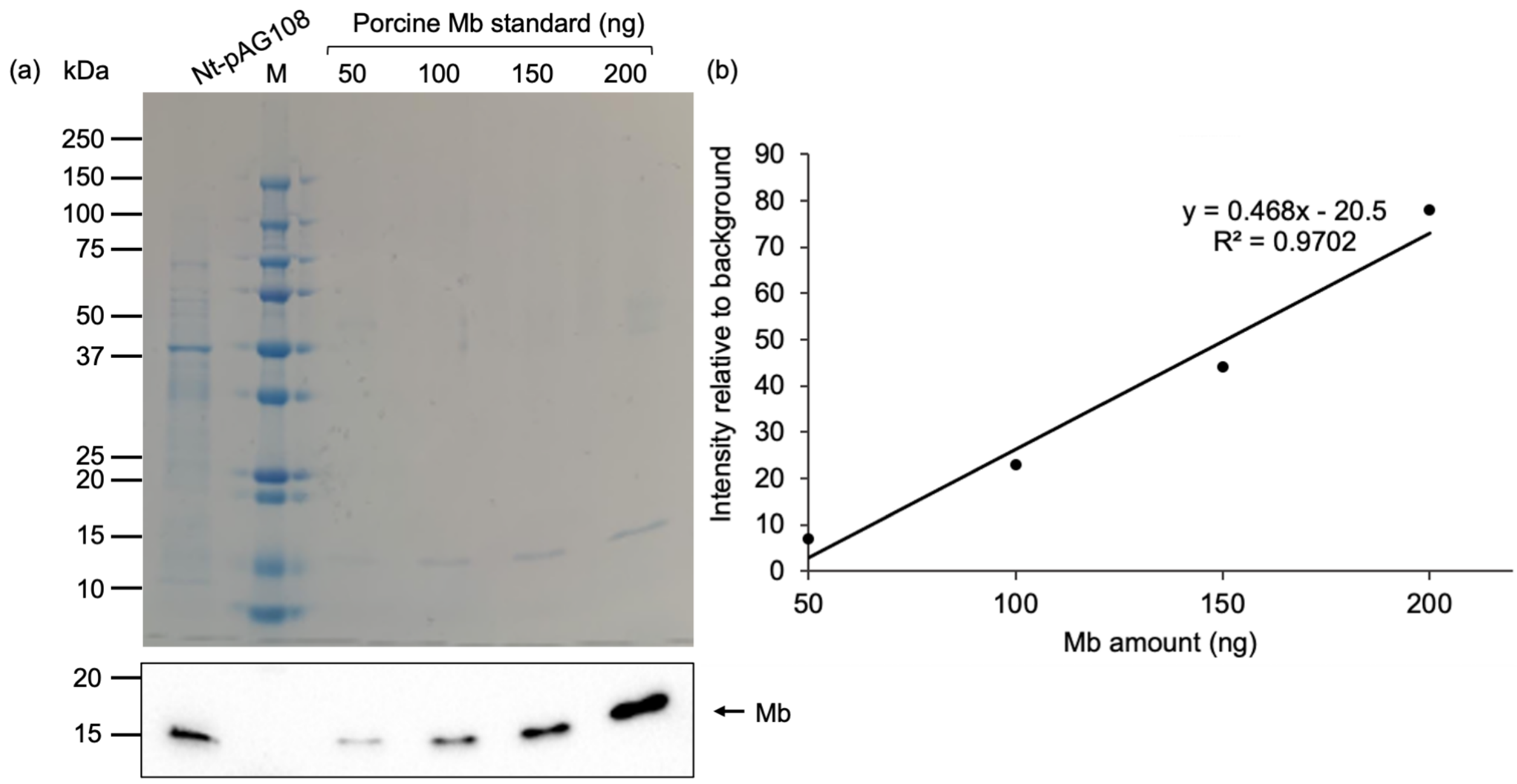

Figure S6. Immunoblot analysis of tobacco Mb-expressing transformant. (a) Immunoblot of total soluble proteins (bottom) using an α-Mb antibody confirms Mb expression and Mb standards (50, 100, 150, 200 ng) allow for quantification of Mb expression by densitometry in tobacco as a proportion of TSP (5 µg total soluble protein loaded). The absence of Nt-wt band is not shown here on this immunoblot but can be observed in Figure 3. The replicate stained SDS-PAGE gel (top) is shown. (b) The immunoblot signals of the standard Mb bands from (a) were measured relative to background for semi-quantitative quantification by densitometry of porcine Mb expression. The tobacco band signal corresponded to 130 ng (2.6% TSP).

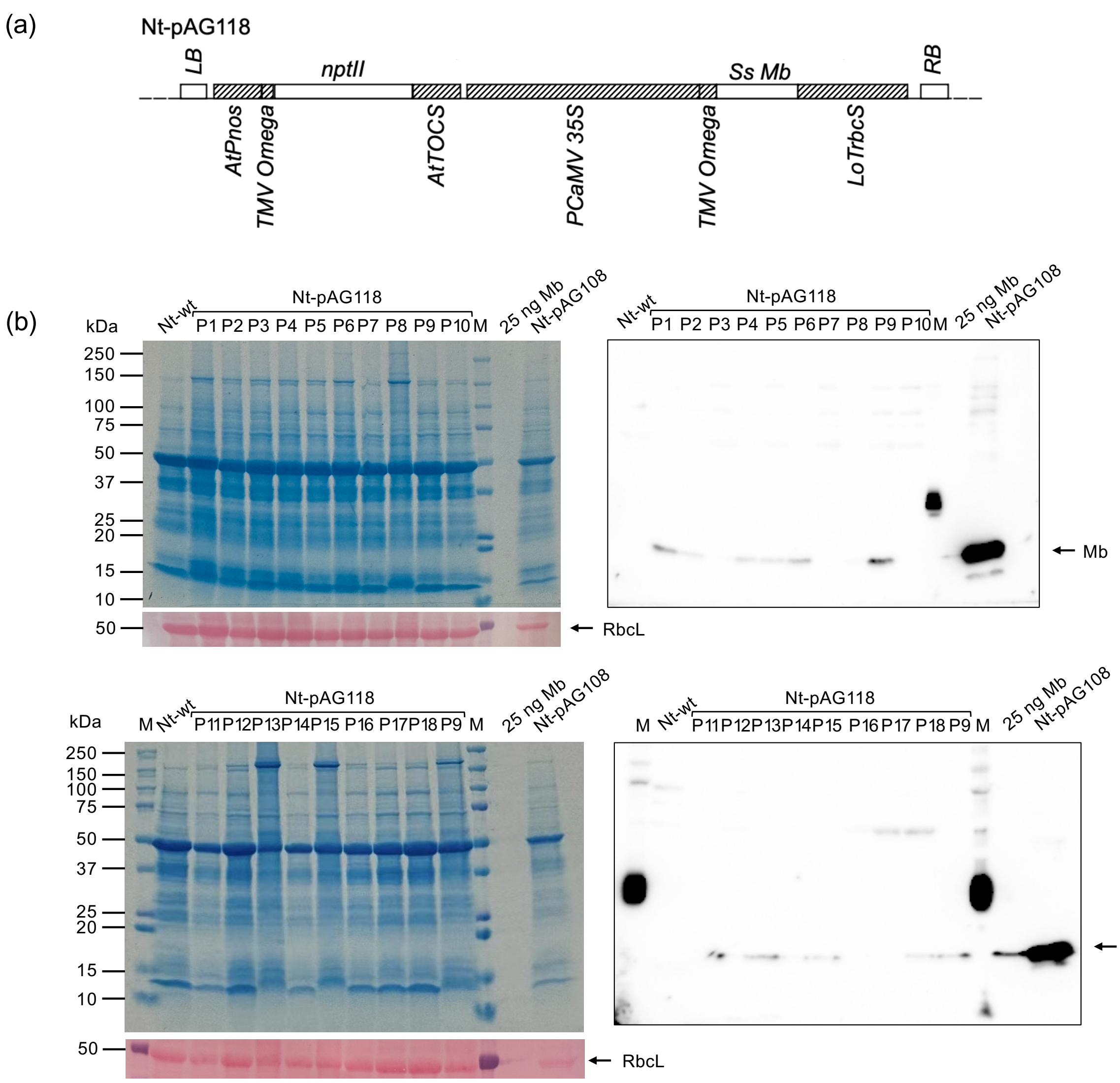

Figure S7. Generation of tobacco nuclear transformants using vector pAG118 and analysis of mutant lines. (a) Map of random insertion within the tobacco nuclear genome and the design of vector pAG118 used for tobacco nuclear transformation. The selectable marker gene *nptII* is driven by the nopaline synthase promoter (Pnos) from *Agrobacterium tumefaciens* and the 5' leader sequence of tobacco mosaic virus RNA (TMV Omega). It is followed by the 3' UTR from the octopine synthase terminator (TOCS) from *Agrobacterium tumefaciens.* The porcine myoglobin (Mb) gene is driven by the cauliflower mosaic virus 35S promoter (PCaMV 35S) and TMV Omega and followed by the 3' UTR from the small subunit of RuBisCO (TrbcS). (b) Immunoblot of soluble fractions of nuclear and transplastomic tobacco lines. Western blotting using an α-Mb antibody (right) allows for Mb detection in nuclear transformant lines (Nt-pAG118) and comparison to the transplastomic mutant Nt-pAG108. The replicate stained SDS-PAGE gel (left) shows loading. Ponceau S staining (bottom) also shows loading judged by the staining of the large RuBisCO subunit (RbcL).

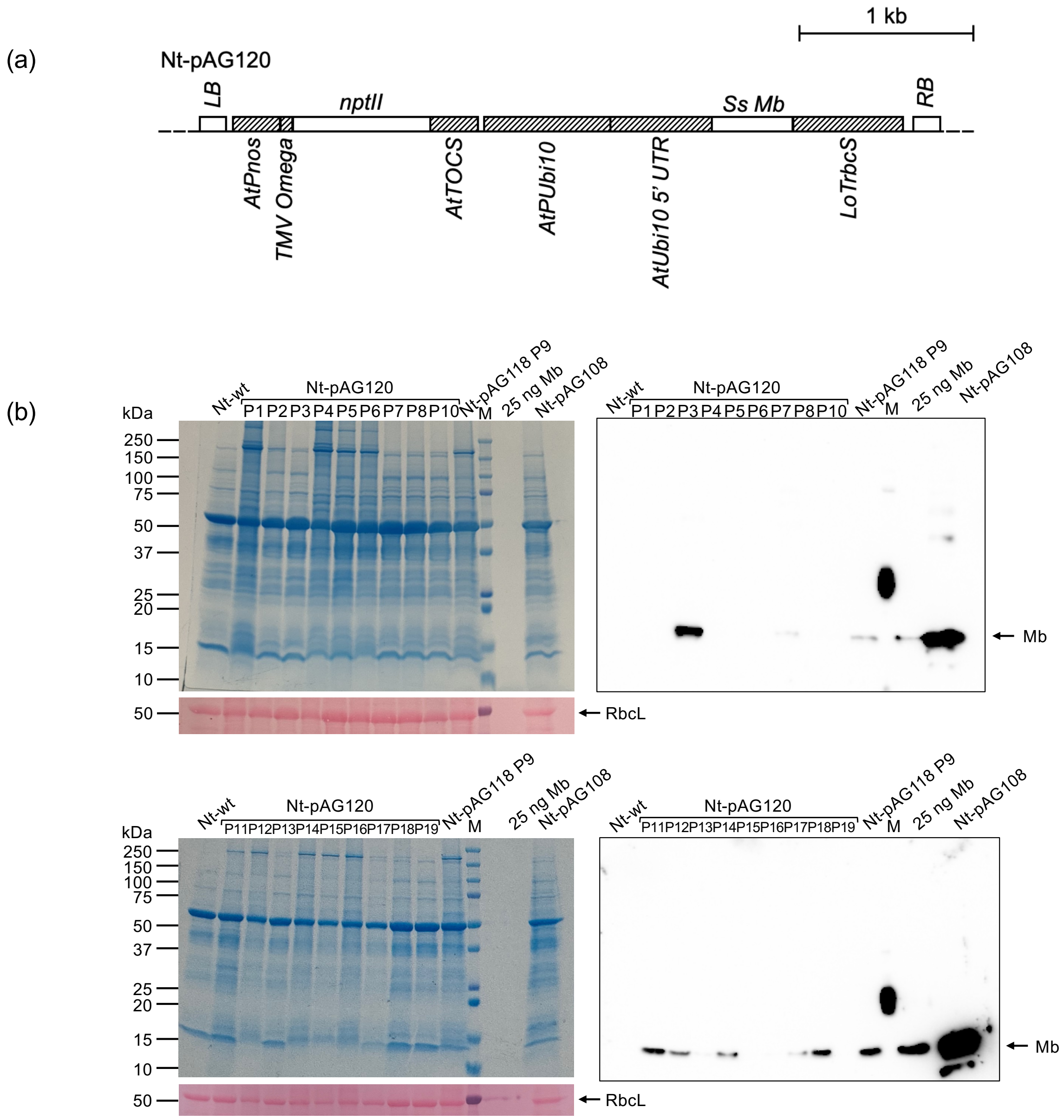

Figure S8. Generation of tobacco nuclear transformants using vector pAG120 and analysis of mutant lines. (a) Map of random insertion within the tobacco nuclear genome and the design of vector pAG120 used for tobacco nuclear transformation. The selectable marker gene *nptII* is driven by the nopaline synthase promoter (Pnos) from *Agrobacterium tumefaciens* and the 5' leader sequence of tobacco mosaic virus RNA (TMV Omega). It is followed by the 3' UTR from the octopine synthase terminator (TOCS) from *Agrobacterium tumefaciens.* The porcine myoglobin (Mb) gene is driven by the *Arabidopsis thaliana* ubiquitin 10 promoter (PAtUbi) and 5' UTR (AtUbi 5' UTR) and followed by the 3' UTR from the small subunit of RuBisCO (TrbcS). (b) Immunoblot of soluble fractions of nuclear and transplastomic tobacco lines. Western blotting using an α-Mb antibody (right) allows for Mb detection in nuclear transformant lines (Nt-pAG120) and comparison to the transplastomic mutant Nt-pAG108. The replicate stained SDS-PAGE gel (left) shows loading. Ponceau S staining (bottom) also shows loading judged by the staining of the large RuBisCO subunit (RbcL).

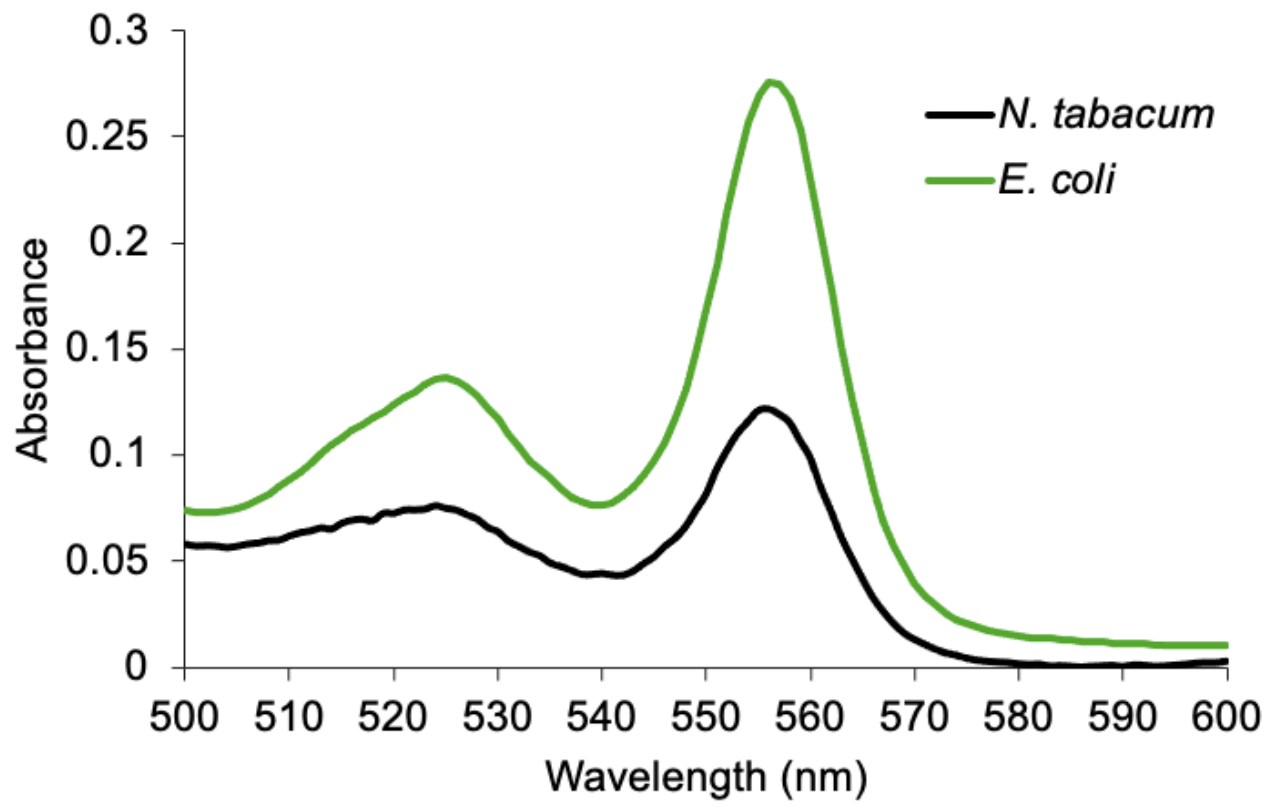

Figure S9. Pyridine hemochromagen assay for purified Mb. Absorbance of reduced pyridine hemochromagen using purified porcine Mb samples. Absorbance of reduced pyridine hemochromagen from tobacco-expressed porcine Mb is measured from 500-600 nm (black); *E. coli*-expressed Mb was included for comparison (green). The protein present in the assay was 10 μM.

Table S1. List of plasmids used in this study.

Plasmid name	Source	Description and use
pAG101	Prof. Peter Nixon	Level 1 Golden Gate vector for bovine Mb expression cassette
pAG102	This work	Transformation vector for Mb expression in *C. reinhardtii*
pAG108	This work	Transformation vector for Mb expression in tobacco
pAG115	This work	Transformation vector for Mb expression in lettuce
pAG112	This work	Transformation vector for Mb expression in *E. coli*
pHJ198	Prof. Saul Purton, Dr. Henry Taunt	Homology arm for inverted repeat region of *C. reinhardtii* chloroplast genome
pHJ192	Prof. Saul Purton, Dr. Henry Taunt	Backbone expression vector for chloroplast expression in *C. reinhardtii*
pHJ205	Prof. Saul Purton, Dr. Henry Taunt	Homology arm for inverted repeat region of *C. reinhardtii* chloroplast genome
pICH51266	Dr. Jiorgos Kourelis	CaMV 35S promoter for nuclear expression in tobacco
pICSL12015	Dr. Jiorgos Kourelis	*Arabidopsis thaliana* ubiquitin promoter for nuclear expression in tobacco
pICSL60004	Dr. Jiorgos Kourelis	*Lathyrus oleraceus* RbcS terminator for nuclear expression in tobacco
pJK403	Dr. Jiorgos Kourelis	Backbone expression vector for nuclear expression in tobacco
pKM014	Kyomei Ltd	Bovine Mb transformation vector for tobacco; backbone for pAG108
pKM058	Kyomei Ltd	Bovine Mb transformation vector for lettuce; backbone for pAG115
pKP9	Prof. Ralph Bock	Empty vector control (*aadA* only) for tobacco chloroplast transformation
pSS117	Prof. Saul Purton, Dr. Henry Taunt	Backbone vector for *C. reinhardtii* vector transformation in *E. coli*
pHT463	Dr. Henry Taunt	Drop-in *E. coli* expression plasmid

Table S2. List of sequences for codon-optimised genes.

Gene (optimisation)	Sequence (5' to 3')	Source plasmid(s)
Bovine Mb (tobacco chloroplast)	ATGGGATTATCTGATGGAGAATGGCAATTAGTATT<br>AAATGCTTGGGGAAAAGTTGAAGCTGATGTTGCT<br>GGTCATGGACAAGAAGTATTAATTAGACTTTTTAC<br>TGGACATCCTGAAACATTAGAAAAATTCGATAAA<br>TTCAAACATCTTAAAACTGAAGCTGAGATGAAAG<br>CTAGTGAAGATCTTAAAAAACATGGAAATACTGT<br>ACTTACAGCTTTAGGTGGAATTTTAAAGAAAAAA<br>GGTCATCATGAAGCTGAAGTTAAACATCTTGCTGA<br>ATCTCATGCTAATAAGCATAAAATTCCTGTTAAAT<br>ATCTTGAATTCATTTCTGATGCTATTATTCATGTTC<br>TTCATGCTAAACATCCTTCTGATTTTGGTGCTGAT<br>GCTCAAGCTGCTATGTCTAAGGCTTTAGAATTGTT<br>TCGTAATGATATGGCAGCTCAATATAAAGTTTTGG<br>GATTTCATGGATAA	pKM014
Porcine Mb (tobacco chloroplast)	ATGGGATTATCTGATGGAGAATGGCAATTAGTATT<br>AAATGTATGGGGAAAAGTAGAAGCTGATGTAGCT<br>GGACATGGACAAGAAGTATTAATTAGATTATTTA<br>AAGGACATCCTGAAACTTTAGAAAAATTTGATAA<br>ATTTAAACATTTAAAATCTGAAGATGAAATGAAA<br>GCTTCTGAAGATTTAAAAAAACATGGAAATACTG<br>TATTAACTGCTTTAGGAGGAATTTTAAAAAAAAA<br>AGGACATCATGAAGCTGAATTAACTCCTTTAGCTC<br>AATCTCATGCTACTAAACATAAAATTCCTGTAAAA<br>TATTTAGAATTTATTTCTGAAGCTATTATTCAAGT<br>ATTACAATCTAAACATCCTGGAGATTTTGGAGCTG<br>ATGCTCAAGGAGCTATGTCTAAAGCTTTAGAATTA<br>TTTAGAAATGATATGGCTGCTAAATATAAAGAATT<br>AGGATTTCAAGGATAA	pAG108
Porcine Mb (lettuce chloroplast)	ATGGGATTATCTGATGGAGAATGGCAATTAGTATT<br>AAATGTATGGGGAAAAGTAGAAGCTGATGTAGCT<br>GGACATGGACAAGAAGTATTGATTCGATTGTTTAA<br>AGGACATCCTGAAACTTTGGAGAAGTTTGATAAA<br>TTCAAACATTTGAAATCTGAGGATGAAATGAAAG<br>CTTCTGAAGATTTGAAAAAACATGGAAATACTGT<br>ATTAACTGCTTTAGGAGGAATTTTGAAAAAGAAA<br>GGACATCATGAAGCTGAATTGACTCCTTTGGCTCA<br>GTCTCATGCTACTAAACATAAAATTCCTGTGAAAT<br>ATTTAGAATTTATTTCTGAAGCTATTATTCAAGTA<br>TTACAATCTAAACATCCTGGAGATTTTGGAGCTGA<br>TGCTCAAGGAGCTATGTCTAAAGCTTTAGAATTAT	pAG115

	TTAGAAATGATATGGCTGCTAAATATAAAGAATTAGGATTTCAAGGATAG	
Porcine Mb (tobacco nucleus)	ATGGGACTTTCTGATGGAGAATGGCAACTTGTTCTTAATGTTTGGGGAAAAGTTGAAGCTGATGTTGCTGGACATGGACAAGAAGTTCTTATTAGACTTTTTAAAGGACATCCAGAAACACTTGAAAAATTTGATAAGTTTAAACATCTTAAGTCTGAAGATGAAATGAAAGCTTCTGAAGATCTTAAGAAACATGGAAATACAGTTCTTACAGCTCTTGGAGGAATTCTTAAAAAGAAAGGACATCATGAAGCTGAACTTACACCACTTGCTCAATCTCATGCTACAAAACATAAAATTCCAGTTAAATATCTTGAATTTATTTCTGAAGCTATTATTCAAGTTCTTCAATCTAAACATCCAGGAGATTTTGGAGCTGATGCTCAAGGAGCTATGTCTAAAGCTCTTGAACTTTTTAGAAATGATATGGCTGCTAAGTATAAGGAACTTGGATTTCAAGGATAA	pAG118, pAG120

Table S3. List of oligonucleotides used in this study. Recognition sequences of introduced restriction sites are underlined.

Oligonucleotide name	Sequence (5' to 3')	Description and use
AG122	CGCACCTGCAACGCATGGGTATATCTCCTTCTG	Amplification of pKM014 backbone
AG123	GGCACCTGCGGATTAATCTAGAGAAATTCAATTAAGGAAATAAATT	
AG134	TGGCTCTTCTAGTGGATTATCTGATGGAGAATGG	Amplification of Mb gene from pAG108
AG135	TCGCTCTTCTTTATTATCCTTGAAATCCTAATTCTTTATATTTAG	
AG145	AGGGCCTTTATGACACAA	Lettuce southern blot probe
AG212	AGCATTTAACCAACCCG	
AG156	CCCACCTGCCGCCATAGTCTAGAGAAATTCAATTAAGGAAATAAAT	Amplification of pKM058 backbone
AG157	GGCACCTGCATCGCCAGAGTAAAACATACAATTTTTTTGAC	
AG208	CCCAGAAAGAGGCTG	Tobacco southern blot probe
AG209	CGGGAACTGCCATTC	
HT492	GTTCTAGTGCCAGGGTTAAA	Homoplasmy PCR analysis of *C. reinhardtii* lines
HT493	GGTAGGTTCTGTCACTGAC	
HT494	TACATTTGTACGGCTCCG	
